# Opposing functions of Fng1 and the Rpd3 HDAC complex in H4 acetylation in *Fusarium graminearum*

**DOI:** 10.1371/journal.pgen.1009185

**Published:** 2020-11-02

**Authors:** Hang Jiang, Aliang Xia, Meng Ye, Jingyi Ren, Dongao Li, Huiquan Liu, Qinhu Wang, Ping Lu, Chunlan Wu, Jin-Rong Xu, Cong Jiang

**Affiliations:** 1 State Key Laboratory of Crop Stress Biology for Arid Areas and NWAFU-Purdue Joint Research Center, College of Plant Protection, Northwest A&F University, Yangling, Shaanxi, China; 2 Department of Botany and Plant Pathology, Purdue University, West Lafayette, IN, United States of America; Universite Paris-Sud, FRANCE

## Abstract

Histone acetylation, balanced by histone acetyltransferase (HAT) and histone deacetylase (HDAC) complexes, affects dynamic transitions of chromatin structure to regulate transcriptional accessibility. However, little is known about the interplay between HAT and HDAC complexes in *Fusarium graminearum*, a causal agent of Fusarium Head Blight (FHB) that uniquely contains chromosomal regions enriched for house-keeping or infection-related genes. In this study, we identified the ortholog of the human inhibitor of growth (ING1) gene in *F*. *graminearum* (*FNG1*) and found that it specifically interacts with the FgEsa1 HAT of the NuA4 complex. Deletion of *FNG1* led to severe growth defects and blocked conidiation, sexual reproduction, DON production, and plant infection. The *fng1* mutant was normal in H3 acetylation but significantly reduced in H4 acetylation. A total of 34 spontaneous suppressors of *fng1* with faster growth rate were isolated. Most of them were still defective in sexual reproduction and plant infection. Thirty two of them had mutations in orthologs of yeast *RPD3*, *SIN3*, and *SDS3*, three key components of the yeast Rpd3L HDAC complex. Four mutations in these three genes were verified to suppress the defects of *fng1* mutant in growth and H4 acetylation. The rest two suppressor strains had a frameshift or nonsense mutation in a glutamine-rich hypothetical protein that may be a novel component of the FgRpd3 HDAC complex in filamentous fungi. FgRpd3, like Fng1, localized in euchromatin. Deletion of *FgRPD3* resulted in severe growth defects and elevated H4 acetylation. In contract, the *Fgsds3* deletion mutant had only a minor reduction in growth rate but *FgSIN3* appeared to be an essential gene. RNA-seq analysis revealed that 48.1% and 54.2% of the genes with altered expression levels in the *fng1* mutant were recovered to normal expression levels in two suppressor strains with mutations in *FgRPD3* and *FgSDS3*, respectively. Taken together, our data showed that Fng1 is important for H4 acetylation as a component of the NuA4 complex and functionally related to the FgRpd3 HDAC complex for transcriptional regulation of genes important for growth, conidiation, sexual reproduction, and plant infection in *F*. *graminearum*.

## Author summary

*Fusarium graminearum* is the major causal agent of Fusarium Head Blight, a devastating disease of wheat and barley worldwide. Epigenetic regulation related to histone acetylation is involved in fungal development and invasive growth. Here, we functionally characterized the ortholog of the human inhibitor of growth (ING1) gene in *F*. *graminearum* (*FNG1*) and revealed its role in histone acetylation. By interacting with the FgEsa1 HAT of the NuA4 complex, Fng1 mediated H4 acetylation and was important for growth, conidiation, sexual development and pathogenicity. The *fng1* mutant was unstable and a total of 34 spontaneous suppressors were isolated. Suppressor mutations were identified in four genes. While three of them, *FgRPD3*, *FgSIN3*, and *FgSDS3*, are key components of the Rpd3 HDAC complex, the other one encodes a glutamine-rich protein appeared to be a novel component of the Rpd3 HDAC complex in filamentous ascomycetes. Nevertheless, none of the mutation occurred in components of other HDAC complexes. Most of spontaneous suppressors were still defective in sexual reproduction and plant infection, indicating a stage-specific relationship between Fng1 and the Rpd3 HDAC complex. FgRpd3 and FgSds3 likely co-localized with Fng1 in euchromatin and played a critical role in vegetative growth. Approximately half of the genes with altered expression levels in the *fng1* mutant were recovered to normal expression levels in two suppressor strains with mutations in *FgRPD3* and *FgSDS3*. Most of these genes had no homologs in yeast, suggesting Fng1 and Rpd3 HDAC complex likely regulates genes unique to *F*. *graminearum* and filamentous fungi and with high genetic variations. Taken together, our data showed the functional relationship between Fng1 and the Rpd3 HDAC complex in H4 acetylation and hyphal growth, which has not been reported in other fungi.

## Introduction

The acetylation of conserved lysine (K) residues in the N-terminal tails of core histone proteins is known to affect dynamic chromatin structure and function [1, 2]. In general, hyperacetylation of histones leads to relaxed chromatin structure and active gene expression, whereas hypoacetylation results in condensed chromatin structure and repressed gene transcription [3]. The acetylation level of histones is mediated by histone acetyltransferases (HATs) and histone deacetylase (HDAC). The balancing action of HAT and HDAC enzymes is important for proper cellular function and development [4].

Most HAT enzymes exist in multi-protein complexes, allowing the enzymes to carry out specific functions in the cell. In the budding yeast *Saccharomyces cerevisiae*, histone acetyltransferases Sas3 and Esa1 are the catalytic subunits of the NuA3 (Nucleosome Acetyltransferase of histone H3) and NuA4 (Nucleosome Acetyltransferase of histone H4) HAT complexes, respectively [5–7]. Sas3, a general activator of gene transcription, is required for both the HAT activity and integrity of the NuA3 complex consisting of Yng1, Eaf6, and two other proteins [6]. Deletion of *SAS3* in *S*. *cerevisiae* has no obvious phenotypes but deletion of *SAS3* together with *GCN5*, another HAT associated with the SAGA complex, is synthetic lethal [8]. In the filamentous ascomycete *Magnaporthe oryzae*, *MoSAS3* has no genetic relationship with *MoGCN5* but the *Mosas3* deletion mutant has severe defects in development and pathogenesis [9]. In comparison with the NuA3 complex, the NuA4 complex is more complex and consists of Esa1 HAT and 12 other proteins. In *S*. *cerevisiae*, *ESA1* is the only essential HAT gene and the *esa1*^ts^ mutant is blocked in the cell cycle at restrictive temperatures [10]. Mutations in the catalytic residues of Esa1 result in defects in DNA mismatch repair, chromosome segregation, replication, and TOR signaling. In filamentous fungi, the *ESA1* ortholog is also an essential gene. In *Aspergillus nidulans*, overexpression of *ESA1* resulted in the transcriptional activation of genes involved in secondary metabolism (SM) [11].

Besides containing different HATs, the yeast NuA3 and NuA4 complexes also have two complex-specific proteins with similar sequences and structures. Yng1 and Yng2, two yeast paralogs homologous to human tumor suppressor ING1 (inhibitor of growth 1), are subunits of the NuA3 and NuA4 complexes, respectively. Both of them have a N-terminal ING domain (PF12998) and a C-terminal PHD (plant homeodomain) finger domain [7, 12–14]. While the PHD finger domain recognizes trimethylated histone, the ING domain binds unmodified histone tails. Yng1 mediates the interaction of Sas3 with the nucleosomes and is required for H3 acetylation by NuA3 [13]. The PHD finger of Yng1 promotes the stabilization of the NuA3 complex at chromatins through the interaction of PHD finger and H3K4me3 [15, 16]. Yng2 is required for transcription activation and DNA damage response in *S*. *cerevisiae* [17, 18]. Unlike Esa1, the yeast *yng2* deletion mutant is viable but defective in response to acidic pH and chemical stress and in H4 acetylation and mitotic and meiotic progression [12, 17].

Although paralogous Yng1 and Yng2 perform different roles in histone acetylation and developmental regulation in *S*. *cerevisiae*, phylogenetic analysis showed that only Yng2 orthologs are conserved in yeast and filamentous ascomycetes, including *Fusarium graminearum*. Yng1 appears to be unique to Saccharomycetales yeasts, likely due to a whole genome duplication event. Filamentous ascomycetes also have HAT genes orthologous to Sas3 and Esa1 as well as orthologs of most components of yeast NuA3 and NuA4 complexes.

*F*. *graminearum* is a causal agent of Fusarium head blight, a destructive wheat disease worldwide. This homothallic ascomycete also produces mycotoxins, deoxynivalenol (DON) and zearalenone (ZEA). In *F*. *graminearum*, histone acetyltransferase FgSas3 is essential for DON production and pathogenicity in wheat head infection [19]. Two other HAT genes homologous to yeast *ELP3* and *GCN5* also are important for fungal development and plant infection by regulating the expression of genes involved in these processes [19, 20]. Interestingly, Gcn5 appears to be targeted by phenazine-1-carboxamide, a compound secreted by the biocontrol agent *Pseudomonas piscium* to inhibit HAT activities of the SAGA complex and consequently reduces the virulence of *F*. *graminearum* [21]. These studies indicated that histone acetylation plays a critical role in regulating hyphal growth, differentiation, reproduction, secondary metabolism and pathogenesis in *F*. *graminearum*.

Although several HAT genes have been functionally characterized, the role of ING protein in histone acetylation and its relationship with HAT complexes are still unknown in *F*. *graminearum*. In this study we found that Fng1, the ortholog of yeast Yng2 in *F*. *graminearum*, is associated with the NuA4 complex to acetylate H4 but dispensable for H3 acetylation. The *fng1* mutant was defective in both development and plant infection, revealing a role of ING proteins in fungal pathogenesis. Suppressor mutations in *FgRPD3*, *FgSIN3*, and *FgSDS3* increased growth rates of the *fng1* mutant but failed to rescue its defects in plant infection and sexual reproduction, suggesting a stage-specific functional relationship between Fng1 and the Rpd3 HDAC complexes. Furthermore, the majority of genes with recovered expression levels by suppressor mutations in *FgRPD3* and *FgSDS3* appear to be unique to *F*. *graminearum* and other filamentous fungi. Taken together, Fng1 is required for the function of the NuA4 complex in H4 acetylation and is genetically related to the Rpd3 HDAC complex for transcriptional regulation of genes important for vegetative growth, conidiation, sexual reproduction, and plant infection in *F*. *graminearum*.

## Results

### Fng1 is specifically associated with the NuA4 complex and involved in H4 acetylation

**Fig 1:**
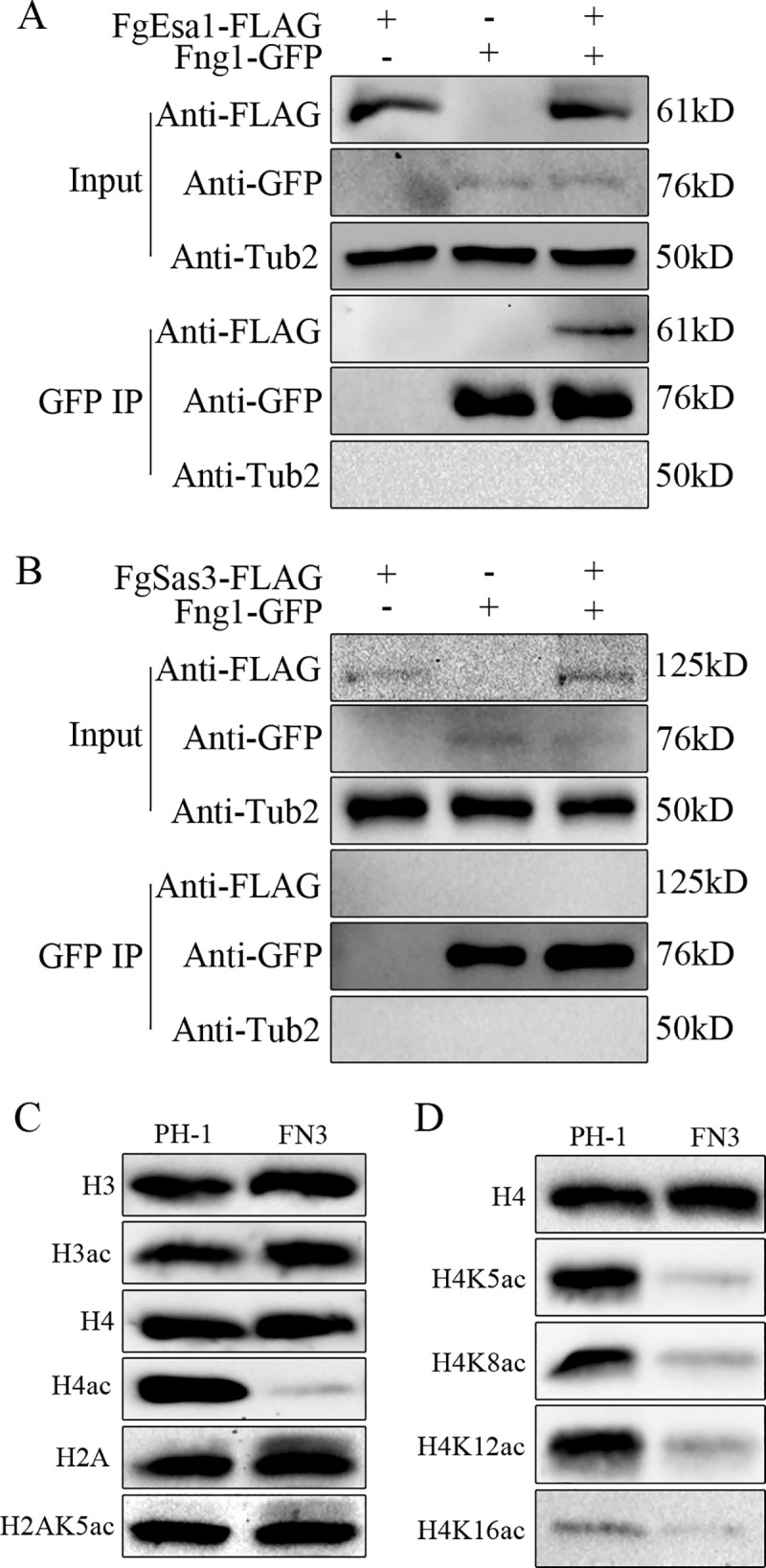
Fng1 is associated with FgEsa1 and required for H4 acetylation. **(A).** Co-immunoprecipitation (co-IP) assays for the interaction between Fng1 and FgEsa1. Western blots of total protein isolated from transformants expressing the *FNG1*-GFP and/or *FgESA1*-3×FLAG (input) and proteins eluted from anti-GFP affinity beads (GFP IP) were detected with anti-FLAG and anti-GFP antibodies. (**B).** Co-IP assays for the interaction between Fng1 and FgSas3. Western blots of total protein isolated from transformants expressing the *FNG1*-GFP and/or *FgSAS3*-3×FLAG (input) and proteins eluted from anti-GFP affinity beads (GFP IP) were detected with anti-FLAG and anti-GFP antibodies. (**C).** Western blots of total proteins isolated from the wild type (PH-1) and *fng1* mutant (FN3) were detected with the anti-H4ac, anti-H3ac, and H2AK5ac antibodies. Detection with the anti-H4, anti-H3, or anti-H2A antibodies was used as loading control. (**D).** Western blots of total proteins isolated from PH-1 and the *fng1* mutant were detected with the antibodies specific for H4K5ac, H4K8ac, H4K12ac, and H4K16ac.

In the genome sequence of *F*. *graminearum* strain PH-1 (RR1), the predicted gene FGRAMPH1_01G03341 encodes a protein with the N-terminal ING (PF12998) and C-terminal PHD (PF00628) domains based on Pfam database (http://pfam.xfam.org/) (S1 Fig) [22]. It was named *FNG1* for *F*. *graminearum* ING1 ortholog 1 in this study. Fng1 is orthologous to yeast *YNG2*. In *S*. *cerevisiae*, Yng1 and Yng2, are paralogous subunits of the NuA3 and NuA4 HAT complexes. To determine whether Fng1 functions as Yng1 and/or Yng2, FgSas3 (FGRAMPH1_01G10071) and FgEsa1 (FGRAMPH1_01G14849) were identified as the orthologs of yeast Sas3 and Esa1, the histone acetyltransferases in the NuA3 and NuA4 complexes[5, 6], by homologous alignment. *FNG1*-GFP and *FgESA1*-FLAG constructs were individually transformed and co-transformed into the wild-type strain PH-1 to generate PH-1/*FNG1*-GFP (FG1), PH-1/*FgESA1*-3×FLAG (EF1), and PH-1/*FNG1*-GFP *FgESA1*-3×FLAG (FGEF1) transformants. In total proteins isolated from vegetative hyphae of the *FNG1*-GFP *FgESA1*-FLAG transformant (FGEF1) (Table 1) and proteins co-purified with anti-GFP affinity beads, the 61-kD FgEsa1-FLAG band was detected with an anti-FLAG antibody (Fig 1A), indicating the association of Fng1 with FgEsa1 *in vivo*. We also generated the *FgSAS3*-FLAG construct and co-transformed it with *FNG1*-GFP into PH-1. In the resulting *FNG1*-GFP *FgSAS3*-FLAG transformant (FGSF1) (Table 1), the 125-kD FgSas3-FLAG band was detected in total proteins but not in proteins co-purified with anti-GFP affinity beads (Fig 1B). Therefore, Fng1 may not interact with FgSas3 or their interaction may be too weak to be detected by co-immunoprecipitation (co-IP) assays.

**Table 1:** The wild type and transformants of *Fusarium graminearum* used in this study.

Strain	Brief description	Reference
PH-1	Wild-type	[23]
FN3	*fng1* deletion mutant of PH-1	This study
FG1	*FNG1*-GFP transformant of PH-1	This study
SF1	*FgSAS3*-3×FLAG transformant of PH-1	This study
FGSF1	*FNG1*-GFP *FgSAS3*-3×FLAG transformant of PH-1	This study
EF1	*FgESA1*-3×FLAG transformant of PH-1	This study
FGEF1	*FNG1*-GFP *FgESA1*-3×FLAG transformant of PH-1	This study
FC1	*fng1*/*FNG1*-GFP transformant	This study
FCNR1	*fng1*/*FNG1*-GFP *FgNOP1*-RFP transformant	This study
FCPD1	*fng1*/ *FNG1*^ΔPHD^ transformant	This study
RP12	*FgRPD3*^Y248C^ transformant of PH-1	This study
FRP12	*fng1 FgRPD3*^Y248C^ transformant	This study
RF5	*FgRPD3*^Δ480–649^ transformant of PH-1	This study
FRF5	*fng1 FgRPD3*^Δ480–649^ transformant	This study
FCRR1	*fng1*/*FNG1*-GFP *FgRPD3*-RFP transformant	This study
RP2	*Fgrpd3* deletion mutant of PH-1	This study
SIS18	*FgSIN3*^ΔCT^ transformant of PH-1	This study
FSIS18	*fng1 FgSIN3*^ΔCT^ transformant	This study
SD38	*FgSDS3*^N103^ transformant of PH-1	This study
FDS38	*fng1 FgSDS3*^N103^ transformant	This study
SD2	*Fgsds3* deletion mutant of PH-1	This study
SC1	*Fgsds3*/*FgSDS3*-GFP transformant	This study
SCRR1	*Fgsds3*/*FgSDS3*-GFP *FgRPD3*-RFP transformant	This study

To characterize the role of Fng1 in histone acetylation, we generated the *fng1* deletion mutant (Table 1) in the wild-type strain PH-1 (S2 Fig). When assayed for histone acetylation with the anti-H4ac antibody, the *fng1* deletion mutant was significantly reduced in H4 acetylation (Fig 1C) in comparison with the wild type. However, H3 and H2AK5 acetylation were not affected in the *fng1* mutant (Fig 1C). We then assayed the acetylation of H4 with the antibodies specific for H4K5ac, H4K8ac, H4K12ac, and H4K16ac. In comparison with the wild type, the *fng1* mutant was significantly reduced in the acetylation of H4K5, H4K8, and H4K12 (Fig 1D). The acetylation of H4K16 also was reduced in the mutant, but the wild type also had a relatively low level of H4K16ac (Fig 1D). These results indicate that Fng1 is associated with the NuA4 complex and it is important for H4 acetylation but deletion of *FNG1* had no obvious effect on H3 or H2AK5 acetylation.

#### *FNG1* plays a critical role in vegetative growth, conidiation, and sexual reproduction

**Fig 2:**
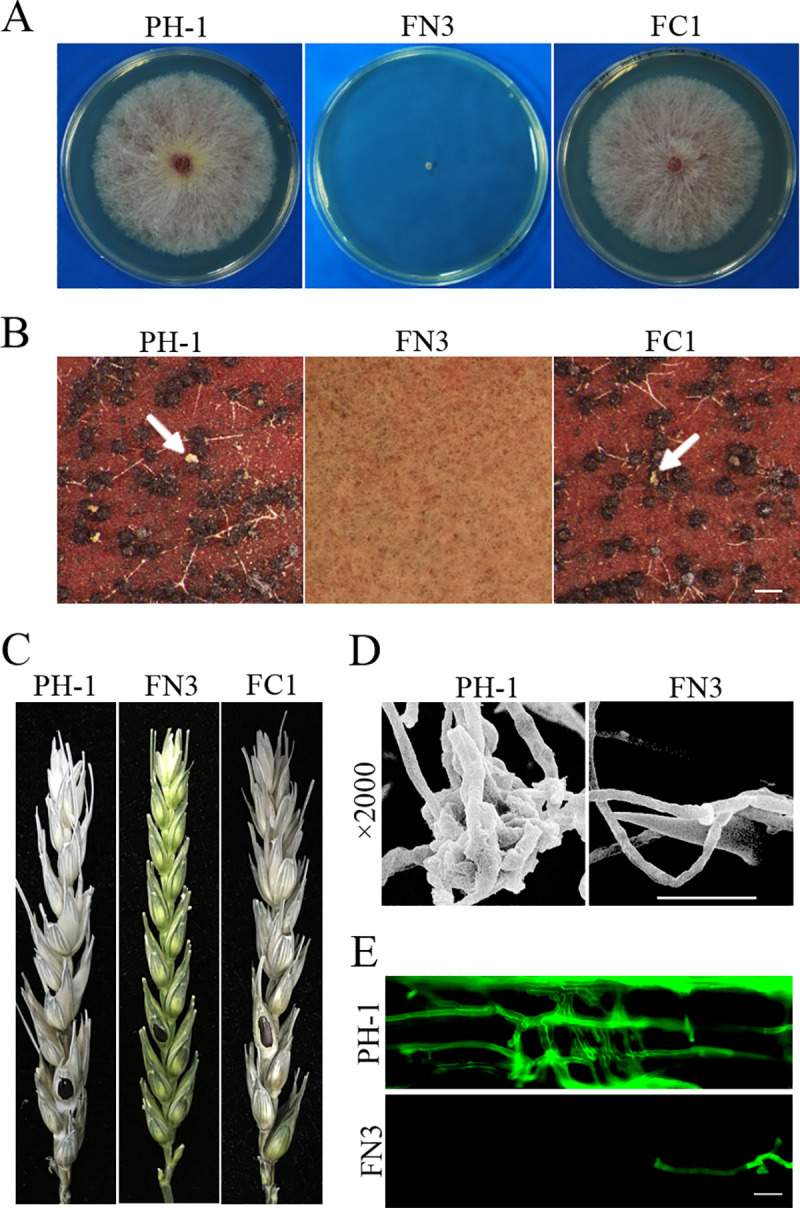
Assays for the function of Fng1 in growth, reproduction, and pathogenesis. **(A).** Three-day-old PDA cultures of the wild type (PH-1), *fng1* mutant (FN3), and *fng1*/*FNG1* transformant (FC1). (**B).** Perithecia from mating cultures of the same set of strains were examined at 8 dpf. Ascospore cirrhi are marked with arrows. Bar, 1 mm. (**C).** Wheat heads inoculated with the indicated strains were examined for head blight symptoms at 14 days post-inoculation (dpi). Black dots mark the inoculated spikelets. (**D).** Infection cushions formed by PH-1 and *fng1* mutant FN3 on wheat lemma were examined by SEM under ×2,000 amplification at 2 dpi. Bar, 20 μm. (**E).** Wheat coleoptiles infected with PH-1 and FN3 were examined for invasive hyphae at 3 dpi after staining with Alexa Fluor 488. Bar, 20 μm.

In comparison with the wild type, the *fng1* deletion mutant was significantly reduced in growth (Table 2) and rarely produced aerial hyphae on PDA plates (Fig 2A). The *fng1* mutant also was defective in asexual and sexual reproduction. In CMC cultures, it failed to produce any conidia (Table 2). In mating cultures on carrot agar plates, the *fng1* mutant failed to form perithecia at 8 days post-fertilization (dpf) or longer (Fig 2B).

**Table 2:** Growth, conidiation, and virulence of the *fng1* mutant and *fng1*/*FNG1*^ΔPHD^ transformant.

Strains	Growth (mm/day)^a^	Conidiation (×10^4^ spores/ml)^b^	Disease Index^c^
PH-1 (WT)	10.9±0.1^A^	20.7±4.2^A^	10.0±1.0^A^
FN3 (*fng1*)	0.6±0.0^C^	nd	nd
FC1 (*fng1*/*FNG1*-GFP)	10.7±0.1^A^	21.3±1.2^A^	10.4±1.1^A^
FCPD1 (*fng1*/*FNG1*^ΔPHD^)	9.6±0.1^B^	8.5±0.5^B^	3.6±0.7^B^

a Average radial growth per day on PDA plates.

b Conidiation in 5-day-old CMC cultures.

c The number of diseased spikelets on each inoculated wheat head at 14 dpi.

The growth rate, conidiation and disease index were assayed with at least three independent replicates. Data were analyzed with Duncan’s pair-wise comparison. Different letters mark significant differences (*P* = 0.05)

nd, not detected

For complementation assays, we generated the *FNG1*-GFP construct and transformed it into the *fng1* mutant. The resulting *fng1*/*FNG1*-GFP transformants were normal in vegetative growth, conidiation and perithecium formation (Fig 2), indicating that the expression of *FNG1*-GFP fully complemented the *fng1* mutant. Therefore, Fng1 is important for vegetative growth, sexual and asexual reproduction.

### *FNG1* is important for infection cushion formation and infectious growth

In infection assays with wheat heads, the *fng1* deletion mutant failed to cause symptoms on the inoculated kernels (Fig 2C). The *fng1* mutant was defective in DON production (S3 Fig) and the expression of the *TRI4*, *TRI5*, *TRI6*, and *TRI10* genes important for DON biosynthesis [24] (S4 Fig). It also failed to cause discoloration or necrosis on corn silks (S5 Fig). The *fng1/FNG1* complemented transformant was normal in virulence. These results indicate that the *fng1* mutant is non-pathogenic and *FNG1* is essential for plant infection.

To further characterize the defects of *fng1* mutant in plant infection, we examined the formation of infection cushions in infected wheat heads by scanning electron microscopy (SEM). Although the wild-type strain PH-1 developed infection cushions on wheat lemma at 2 days post-inoculation which enable it to penetrate into the plant cell, typical infection cushions were not detected in *fng1* mutant-infected samples (Fig 2D), indicating that Fng1 plays a crucial role in infection cushion formation. In infection assays with wheat seedlings [25], the *fng1* mutant was only able to infect through wounds in a few samples and had limited infectious growth inside coleoptile cells adjacent to the wound sites (Fig 2E). While abundant invasive hyphae were produced by the wild type inside plant cells at 3 dpi, extensive spreading of invasive hyphae was not observed in wheat coleoptiles inoculated the *fng1* mutant (Fig 2E). These results indicated *Fng1* may be also important for penetration and infectious growth inside plant tissues.

### Fng1 mainly localizes to euchromatin

**Fig 3:**
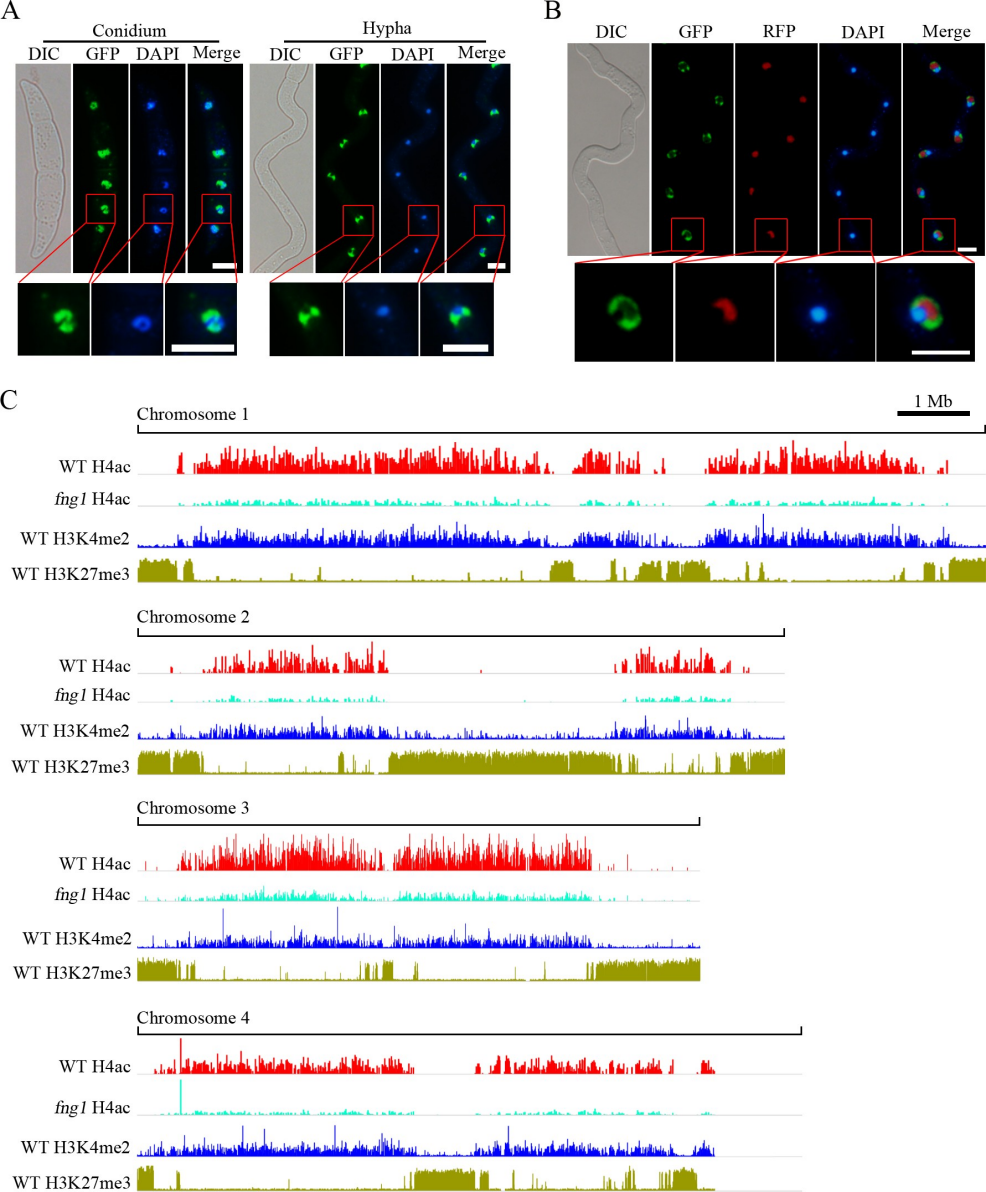
Subcellular localization of Fng1-GFP and chromosomal regions enriched for Fng1-dependent H4ac. (A). Conidia and germlings of the *FNG1*-GFP transformant were stained with DAPI and examined by DIC and epifluorescence microscopy. Bar, 5 μm. (B). Germlings of the *FNG1*-GFP *FgNOP1*-RFP transformant (FCNR1) were stained with DAPI and examined by DIC and epifluorescence microscopy. Bar, 5 μm. The lower panels are close-up view of the indicated nuclei. (C). The distribution of DNA sequences co-immunoprecipitated with the anti-H4ac antibody in the wild-type strain PH-1 and *fng1* mutant (chromosomes 1–4) was compared with chromosomal regions enriched for H3K27me3 and H3K4me2 in the genome of *F*. *graminearum*.

To determine the localization of Fng1, we transformed the *FNG1*-GFP fusion construct into the *fng1* mutant. In the resulting *fng1*/*FNG1*-GFP transformants (Table 1), GFP signals were observed in the nucleus in conidia and vegetative hyphae (Fig 3A), which is consistent with its function as a component of the NuA4 HAT complex in *F*. *graminearum*. However, because GFP signals were unevenly distributed in the nucleus (Fig 3A), we generated and transformed the *FgNOP1*-RFP construct into the *fng1*/*FNG1*-GFP transformant FC1. Nop1 is a marker for the nucleolus [26]. When the *FNG1*-GFP *FgNOP1*-RFP transformant (FCNR1) (Table 1) was stained with DAPI and examined by epifluorescence microscopy, Fng1-GFP signals were consistently observed adjacent to the FgNop1-RFP signals in the nucleus but they did not overlap (Fig 3B). Fluorescent signals of DAPI-stained DNA also rarely overlapped with Fng1-GFP and FgNop1-RFP signals (Fig 3B). Because the regions stained strongly with DAPI correspond to centromeric heterochromatins [27], our data suggested that Fng1 and its associated NuA4 complex are likely enriched in euchromatin.

A previous study has reported that H3K4me2 and H3K27me3 are associated with euchromatin and heterochromatin, respectively, in *F*. *graminearum* [28]. To verify the association between Fng1 and euchromatin, ChIP-seq data were generated with the anti-H4ac antibody for the wild type and *fng1* mutant. Genome-wide comparison showed that H4ac enrichment was significantly reduced or almost lost in the *fng1* mutant compared to PH-1 (Fig 3C). Chromosomal regions enriched for Fng1-dependent H4ac had a similar distribution pattern with that of H3K4me2, which is mutually exclusive of H3K27me3 (Fig 3C). These results suggested that Fng1-mediated H4 acetylation is associated with euchromatin.

### The PHD finger domain is important but not essential for the function of Fng1

**Fig 4:**
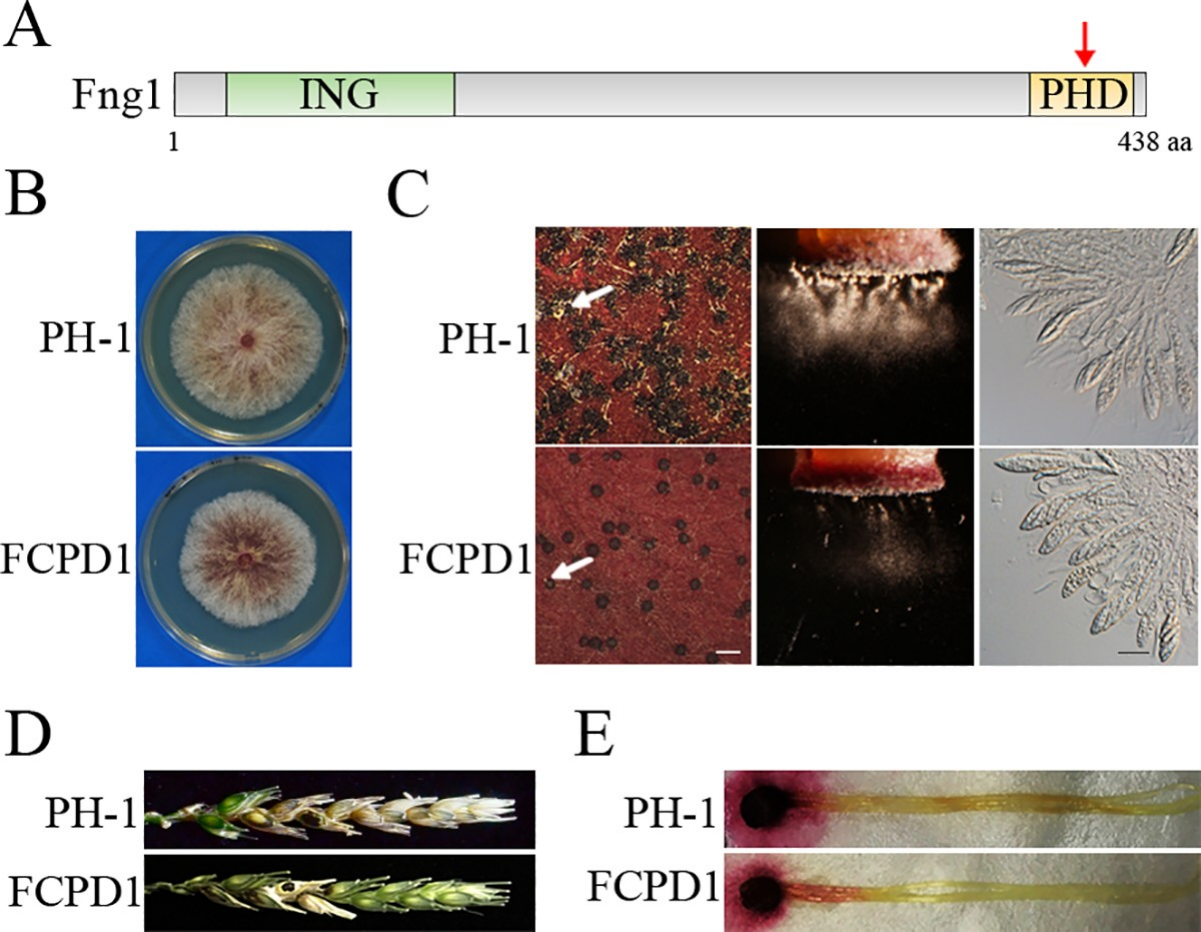
The effects of PHD domain deletion on *FNG1* functions. **(A).** Schematic drawing of the Fng1 protein with the ING and PHD domains indicated by red arrow. (**B).** Three-day-old PDA cultures of PH-1 and *fng1*/*FNG1*^ΔPHD^ transformant (FCPD1). (**C).** Mating cultures were examined for perithecium formation (left), ascospore discharge (middle), and asci with ascospores (right) at 8 dpf. Ascospore cirrhi are indicated by arrows. White bar, 1 mm; Black bar, 20 μm. (**D).** Wheat heads inoculated with the indicated strains were examined for head blight symptoms at 14 dpi. Black dots mark the inoculated spikelets. (**E).** Corn silks inoculated with culture blocks were photographed at 5 dpi.

The PHD finger domain present in many chromatin-associated proteins is known to bind with trimethylated lysines on histone tails [16]. To determine the function of the C-terminal PHD domain in *FNG1*, we generated the *FNG1*^ΔPHD^ construct deleted of residues 386–433 (Fig 4A, S6 Fig) and transformed it into the *fng1* mutant. The resulting *fng1*/*FNG1*^ΔPHD^ transformant FCPD1 (Table 1) grew slower than the wild-type strain PH-1 on PDA (Fig 4B) and was reduced in conidiation (Table 2). It produced fewer perithecia with normal asci and ascospores on mating plates than the wild type and formed fewer ascospore cirrhi (Fig 4C). Deletion of the PHD domain in *FNG1* also affected forcible discharge of ascospores (Fig 4C). In infection assays with flowering wheat heads, the *fng1*/*FNG1*^ΔPHD^ transformant caused typical scab symptoms in the inoculated wheat kernels and was able to spread to nearby spikelets (Fig 4D). Nevertheless, its virulence was reduced by 70% compared with PH-1 (Table 2). These results indicate that the PHD finger domain is important but not essential for the function of Fng1.

### Spontaneous suppressors of the *fng1* mutant

**Fig 5:**
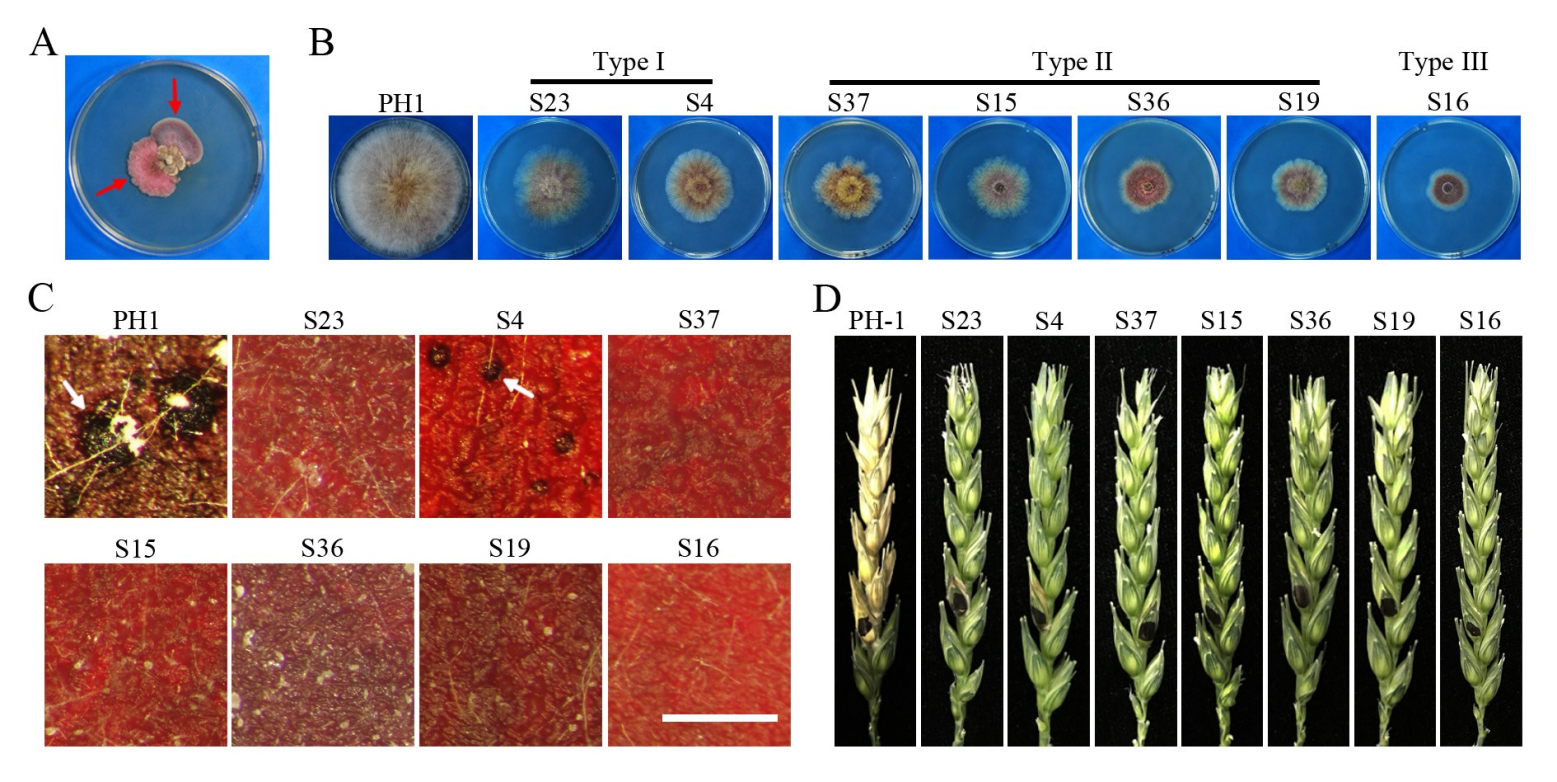
Spontaneous suppressors of the *fng1* mutant. **(A).** PDA cultures of the *fng1* mutant strains after incubation for more than two weeks. Suppressor strains are indicated by the red arrows. (**B).** Four-day-old PDA cultures of the wild type (PH-1) and representative suppressor mutants which were divided into three types based on growth rate. (**C).** Mating cultures of PH-1 and indicated suppressor strains were examined at 8 dpf. Bar, 1 mm. Perithecia are indicated by arrows. (**D).** Flowering wheat heads were inoculated with PH-1 and indicated suppressor strains, and photographed at 14 dpi. Black dots mark the inoculated spikelets.

The *fng1* mutant was unstable and often formed fast-growing sectors on the edge of colonies that had limited growth on PDA plates after incubation for two weeks (Fig 5A). A total of 34 fast-growing sectors were isolated as spontaneous suppressor mutants and categorized into three groups based on average radial growth per day (S1 Table). While eight type I suppressor strains had the fastest growth (>50% of the wild type) and wild-type colony morphology, growth of 15 type II suppressor strains recovered to 35–50% of that of the wild type. The remaining 11 Type III suppressor strains had the slowest growth rate but still grew faster than the *fng1* mutant (Fig 5B, S1 Table).

Besides changes in growth rate and colony morphology, all the suppressor mutants were assayed for phenotypes in conidiation, sexual reproduction, plant infection and DON production (Fig 5C and 5D, S1 Table). Most of the suppressor strains, except S22 and S32, produced conidia, but conidiation was reduced in comparison with the wild type (S1 Table). While most of the suppressor mutants were still blocked in perithecium formation, three suppressor mutants (S4, S12 and S29) formed smaller perithecia that lacked asci or ascospores (Fig 5C, S1 Table). In infection assays with wheat heads, similar to the original *fng1* mutant, most suppressor strains were non-pathogenic. However, suppressor strains S4, S12, S23, S24, S26, S31, S34, S47, S49 and S52 caused discoloration on the inoculated kernel but failed to spread to neighboring spikelets (Fig 5D, S1 Table). While majority of these suppressor strains still failed to produce DON in LTB (liquid trichothecene biosynthesis) cultures, eight of them (S24, S25, S26, S31, S33, S36, S38, and S46) were partially recovered in DON biosynthesis (S1 Table). These results suggested that those suppressor mutations resulting in faster growth rate often partially rescued conidiation defects of the *fng1* mutant. However, none of them resulted in ascospore or ascus development and spreading in infected wheat heads although some of them also rescued the defects of the *fng1* mutant in early stage of sexual reproduction and initial plant infection.

### Identification of suppressor mutations in components of the RPD3L HDAC complex

**Fig 6:**
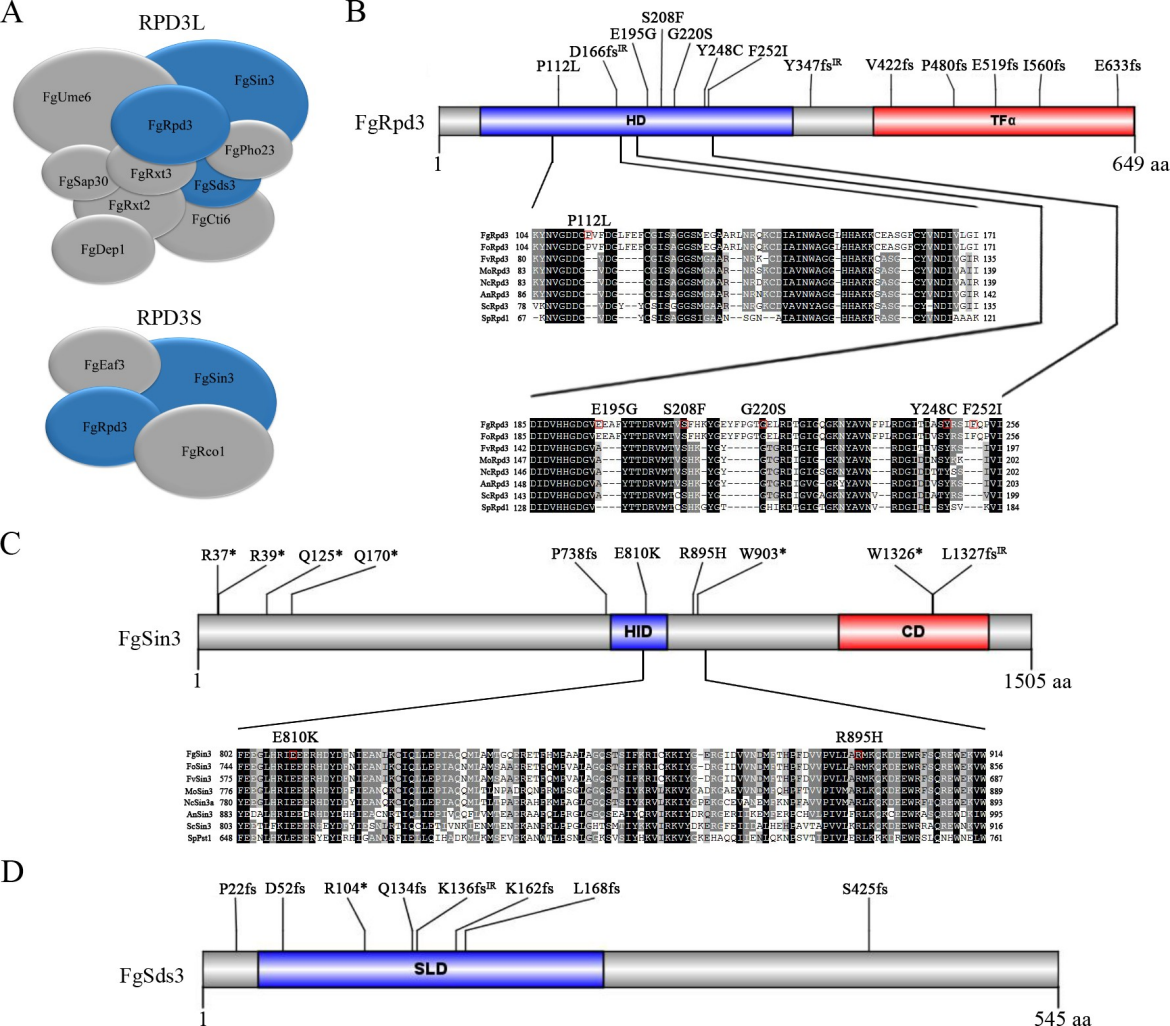
Suppressors identified in the RPD3 HDAC complex. **(A).** Schematic diagrams showing components of the yeast Rpd3L and Rpd3S complexes with orthologs in *F*. *graminearum*. (**B).** Spontaneous suppressor mutations identified in FgRpd3. The histone deacetylase (HD) domain and transcription initiation factor IIF-alpha (TFα) domain are shaded in blue and red. Sequence alignments of the marked region of FgRpd3 with its orthologs from *F*. *oxysporum* (Fo), *F*. *verticillioides* (Fv), *M*. *oryzae* (Mo), *N*. *crassa* (Nc), *A*. *nidulans* (An), *S*. *cerevisiae* (Sc), and *S*. *pombe* (Sp). (**C).** Schematic drawing of FgSin3 and spontaneous suppressor mutations. Sequence alignments of the marked region of FgSin3 with its orthologs from Fo, Fv, Mo, Nc, An, Sc, and Sp. HID, histone deacetylase interacting domain; CD, C-terminal domain. **(D).** Schematic drawing of FgSds3 and spontaneous suppressor mutations. SLD, Sds3-like domain.

The original *fng1* mutant and four suppressor strains (S18, S19, S32 and S38) were selected for genome sequencing. Suppressor mutations were identified in *FgRPD3* (FGRAMPH1_01G01959), *FgSIN3* (FGRAMPH1_01G27415), *FgSDS3* (FGRAMPH1_01G02071) and FGRAMPH1_01G22839 (S2 Table). We then amplified and sequenced these four genes in the remaining 30 suppressor strains, and found that another 12, 9, 8, and 1 of them had mutations in the *FgRPD3*, *FgSIN3*, *FgSDS3*, and FGRAMPH1_01G22839 genes, respectively (Table 3). Interestingly, FgRpd3, FgSin3, and FgSds3 are the key subunits of the Rpd3 histone deacetylase (HDAC) complexes (Fig 6A). In *S*. *cerevisiae*, the histone deacetylase Rpd3 resides in the Rpd3L (large) and Rpd3S (small) HDAC complexes that differ in functions [29]. Rpd3 and Sin3 belong to both Rpd3L and Rpd3S complexes, but Sds3 is a Rpd3L-specific subunit [30]. Because multiple suppressor strains had mutations in *FgSDS3* and they were similar to suppressor strains with mutations in *FgRPD3* and *FgSIN3* in colony morphology or growth rate, it is likely that mutations in the FgRpd3L HDAC complex were suppressive to the *fng1* mutant. Therefore, it is possible that only the RPD3L HDAC complex is associated with the function of Fng1 and NuA4 HAT complex in *F*. *graminearum*.

**Table 3:** Mutations identified in suppressor strains.

	Suppressor strains	Nucleotide change	Amino acid changes
FGRAMPH1_01G01959 (*FgRPD3*)	S1^#^	AG^615^ to AA (intron 2)	fs ^IR^
	S4	T^873^TC to ATC	F252I
	S5	ΔC^1606^	P480fs
	S10	TC^742^T to TTT	S208F
	S11	G^777^GT to AGT	G220S
	S12	TA^862^C to TGC	Y248C
	S16	GAT^1430^ to GATT	V422fs
	S19	CC^407^T to CTT	P112L
	S29	AA^1195^ to AC (intron 3)	fs ^IR^
	S44	ΔGAGA^2064-2067^	E633fs
	S47	GA^703^G to GGG	E195G
	S50	AAC^1843^ to AACAC	I560fs
	S52	ΔGAGA^1725-1728^	E519fs
FGRAMPH1_01G27415 (*FgSIN3*)	S8	TG^3199^G to TAG	W903*
	S15	C^373^AG to TAG	Q125*
	S18	TG^4468^G to TAG	W1326*
	S23	C^115^GA to TGA	R39*
	S34	C^109^GA to TGA	R37*
	S37	G^4470^T to AT (intron 5)	fs ^IR^
	S40	ΔC^2704^	P738fs
	S41	C^508^AA to TAA	Q170*
	S42	G^2919^AG to AAG	E810K
	S43	CG^3175^T to CAT	R895H
FGRAMPH1_01G02071 (*FgSDS3*)	S24	ΔC^65^	P22fs
	S25	Δ^-794 to -412^	promoter loss
	S26	CAC^560^ to CACC	L168fs
	S31	ΔT^1336^	S425fs
	S33	GGA^155^ to GGAA	D52fs
	S36	ΔCA^400-401^	Q134fs
	S38	C^310^GA to TGA	R104*
	S46	ΔAAGGCTCTTACGAGCAC^544-560^	K162fs
	S49	AG^468^ to AA (intron 1)	fs ^IR^
FGRAMPH1_01G22839	S22	ΔTC^3185-3186^	A1023fs
	S32	C^1413^AA to TAA	Q431*

# Numbered in the order of suppressor strain isolation

* stop codon; fs frame shift; IR, intron retention

In total, 32 suppressor strains had suppressor mutations in *FgRPD3*, *FgSIN3*, and *FgSDS3* genes, including 16 frame-shift, 7 nonsense, and 8 missense mutations (Fig 6B, 6C and 6D). Four of the frame-shift mutations in suppressor strains S1, S29, S37, and S49 had intron splicing defects caused by nucleotide sequence changes at the splicing sites. Suppressor S29 had an A-to-C mutation at the predicted branch point of intron 3 of *FgRPD3*. RT-PCR analysis indicated an intron retention in this suppressor strain S29, suggesting that the splicing efficiency was significantly reduced (S7 Fig). Suppressor S49 had the AG^468^ to AA mutation that affected the splicing of the first intron of *FgSDS3*, resulting in the 60-bp intron retention (S8 Fig). Only two suppressor mutations were not in genes that may be related to the Rpd3L HDAC complex. Suppressor strains S22 and S32 had frameshift and nonsense mutations in FGRAMPH1_01G22839 (Table 3) that lacks homologs in the budding or fission yeast and has no known homolog in GenBank.

Twenty-five suppressor strains with >30% growth rate of the wild-type were selected for correlation analysis between their phenotypes and suppressor mutations. In general, suppressor strains with mutations in *FgRPD3* and *FgSIN3* grew faster than the ones with mutations in *FgSDS3* (S1 Table, S9 Fig). However, there were significant variations among suppressor strains with mutations in the same gene (S1 Table, S9 Fig). For examples, among the five suppressor strains with missense mutations in *FgRPD3*, S47, S4, S12, and S11 had similar growth rate but strain S19 grew much slower (S1 Table). Strain S5 had a slower growth rate than other suppressor strains with frame shift mutations in *FgRPD3* (S1 Table,). Although most of the suppressor mutations in *FgSIN3* were frame-shift mutations, the two suppressor strains with missense mutation in *FgSIN3*, S42 and S43, differed significantly in growth rates and colony morphology (S10 Fig). Interestingly, all the suppressor strains that were partially recovered in DON biosynthesis had mutations in *FgSDS3*.

### The growth defect of *fng1* mutant is partially rescued by the Y248C mutation and C-terminal truncation of FgRpd3

Among the mutations in the *FgRPD3* HDAC gene, all six missense mutations are in the histone deacetylase (HD) domain(PF00850), including the Y248C mutation identified in suppressor S12 (Fig 6B). We also identified six frame-shift mutations resulting in the truncation of the TFα domain (PF05793) at the C-terminus of FgRpd3, including the P480fs mutation identified in suppressor S5. The Y248C and P480fs mutations were selected for verification because sequence alignment revealed that Y248 is well conserved in Rpd3 orthologs and frame-shift mutation at P480 likely affects the function of FgRpd3 by disrupting the TFα domain (Fig 6B).

**Fig 7:**
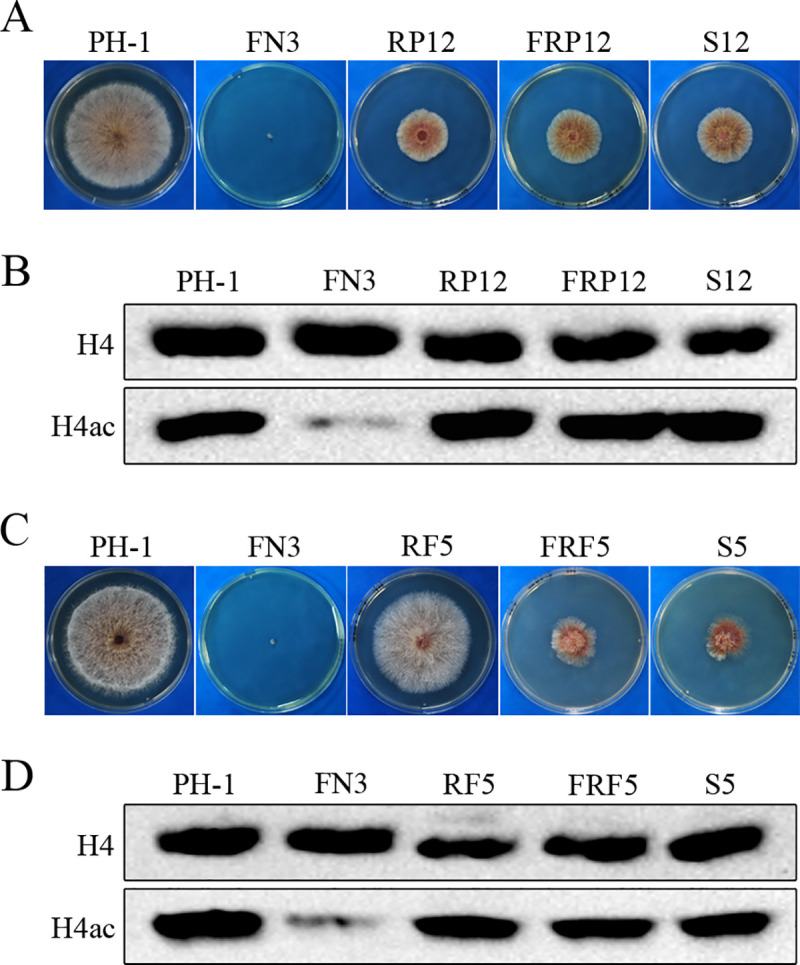
Mutations in *FgRPD3* suppressive to the growth defects of the *fng1* mutant. **(A).** Three-day-old PDA cultures of wild type (PH-1), *fng1* mutant (FN3), *FgRPD3*^Y248C^ (RP12), *fng1 FgRPD3*^Y248C^ (FRP12), and S12. **(B).** Western blots of total proteins isolated from the indicated strains were detected with the anti-H4ac antibody. Detection with the anti-H4 antibody was used as a loading control. **(C).** Three-day-old PDA cultures of PH-1, FN3, *FgRPD3*^Δ480–649^ (RF5), *fng1 FgRPD3*^Δ480–649^ (FRF5), and S5. **(D).** Western blots of total proteins isolated from the indicated strains were detected with the anti-H4ac antibody. Detection with the anti-H4 antibody was used as a loading control.

To determine the effect of Y248C mutation, the *FgRPD3*^Y248C^ mutant allele was generated and used to replace the *FgRPD3* allele. The resulting transformant RP12 (Table 1) grew slower than the wild type (Fig 7A), indicating mutations occurred at Y248 affected the function of FgRpd3 in growth. We then used the gene replacement approach to delete *FNG1* in the *FgRPD3*^Y248C^ transformant RP12. Similar to the suppressor strain S12, the *fng1 FgRPD3*^Y248C^ transformant (Table 1) grew faster than the *fng1* mutant (Fig 7A) and had normal H4 acetylation levels (Fig 7B).

To verify the suppressive effects of these frameshift mutations, we replaced the original *FgRPD3* allele with *FgRPD3*^Δ480–649^ to disrupt the TFα domain. The resulting *FgRPD3*^Δ480–649^ transformant RF5 (Table 1) was only slightly reduced in growth rate (Fig 7C). We also generated the *fng1 FgRPD3*^Δ480–649^ mutant (Table 1) by targeted deletion of *FNG1* in the *FgRPD3*^Δ480–649^ transformant. The *fng1 FgRPD3*^Δ480–649^ mutant had a growth rate (Fig 7C) and H4 acetylation level (Fig 7D) similar to suppressor strain S5. These results indicate that frameshift mutations in FgRpd3 resulting in truncations of the TFα domain had little effect on vegetative growth but partially rescued the growth defects of the *fng1* mutant.

### FgRpd3 co-localizes with Fng1 and negatively impacts H4 acetylation

**Fig 8:**
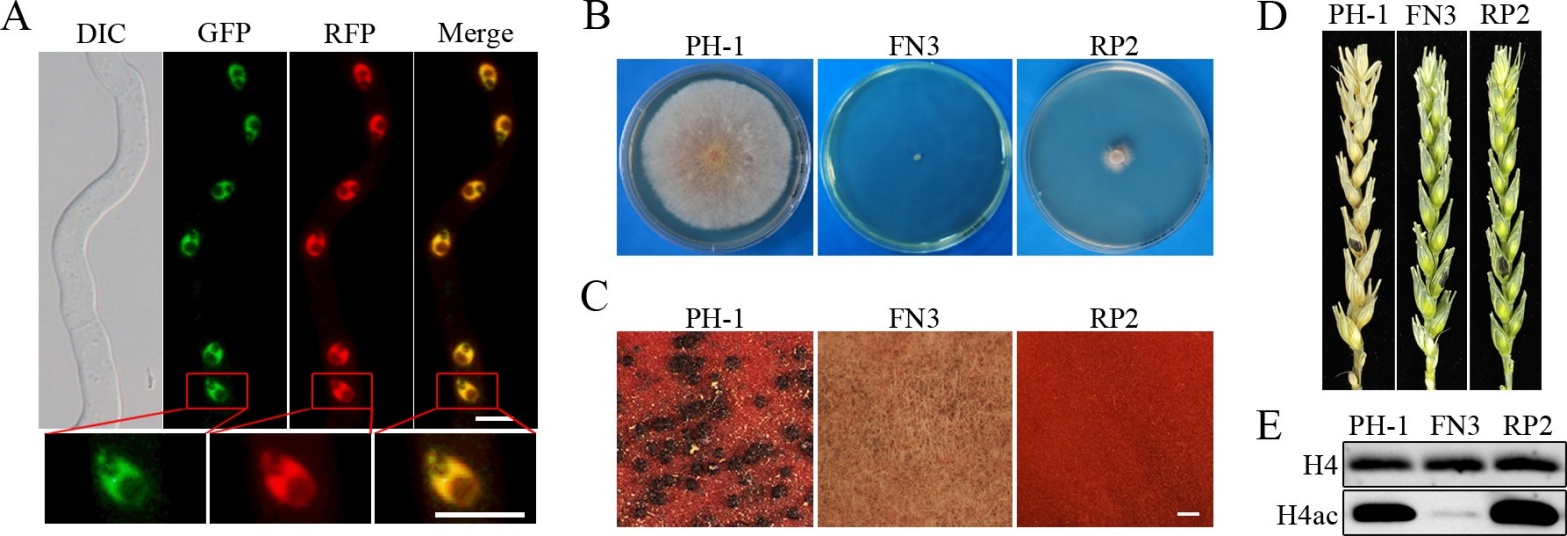
Localization of FgRpd3 and phenotypes of the *Fgrpd3* deletion mutant. **(A).** Germlings of the *FNG1*-GFP *FgRPD3*-RFP transformant (FCRR1) were examined by DIC and epifluorescence microscopy. The lower panels are close-up view of the indicated nuclei. Bar, 5 μm. (**B).** Three-day-old PDA cultures of PH-1, *fng1* mutant (FN3), and *Fgrpd3* mutant (RP2). (**C).** 8 days post-fertilization (dpf) mating cultures of the indicated strains. Bar, 1 mm. (**D).** Flowering wheat heads inoculated with PH-1, FN3, and RP2 were photographed at 14 dpi. Black dots mark the inoculated spikelets. (**E).** Western blots of total proteins isolated from PH-1, FN3, and RP2 were detected with an anti-H4ac antibody. Detection with the anti-H4 antibody was used as a loading control.

To further determine the relationship between Fng1 and FgRpd3, we first generated the *FgRPD3*-RFP fusion construct and transformed it into the *fng1/FNG1*-GFP strain FC1 (Table 1). In the resulting transformant *fng1*/*FNG1*-GFP *FgRPD3*-RFP (FCRR1) (Table 1), both FgRpd3-RFP and Fng1-GFP proteins accumulated in the nucleus (Fig 8A). Close examination of germ tubes in transformant FCRR1 showed that the FgRpd3-RFP and Fng1-GFP signals overlapped in the nucleus, probably in euchromatin (Fig 8A), indicating a high degree of co-localization.

We then generated the *Fgrpd3* deletion mutant by gene replacement (Table 1, S2 Fig). The *Fgrpd3* mutant had severe growth defects (Fig 8B) and failed to form perithecia on mating plates (Fig 8C) and was non-pathogenic in infection assays with wheat heads (Fig 8D). However, the H4 acetylation level was significantly increased in the *Fgrpd3* mutant (Fig 8E), indicating that *FgRPD3* negatively regulates the acetylation of H4.

### Mutations in FgSin3 also suppress the growth defects of the *fng1* mutant

**Fig 9:**
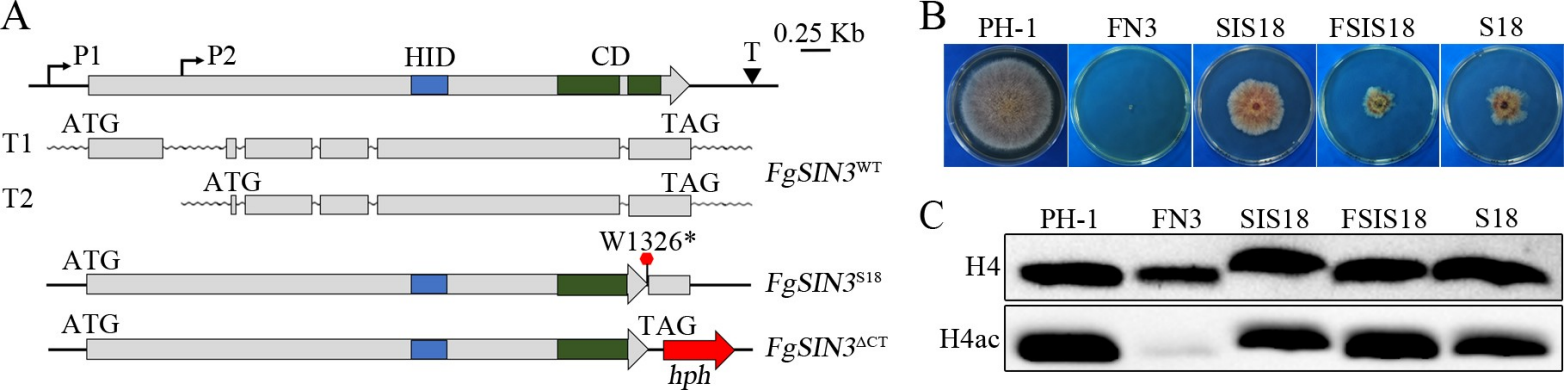
Mutations in *FgSIN3* suppress the growth defect of the *fng1* mutant. **(A).** Schematic drawing of different alleles of *FgSIN3* and its transcripts. The longer (T1) and shorter (T2) transcripts were generated with alternative transcript initiation sites (P1 and P2) but the same termination site (T). While suppressor strain S18 had the non-sense mutation at W1326, the *FgSIN3*^ΔCT^ mutant had the C-terminal 180 amino acid residues replaced with the hygromycin phosphotransferase (*hph*) cassette. HID, histone deacetylase-interacting domain; CD, C-terminal domain. (**B).** Three-day-old PDA cultures of wild type (PH-1), *fng1* mutant (FN3), *FgSIN3*^ΔCT^ (SIS18), *fng1 FgSIN3*^ΔCT^ (FSIS18), and suppressor strain S18. (**C).** Western blots of total proteins isolated from the indicated strains were detected with an anti-H4ac antibody. Detection with the anti-H4 antibody was used as a loading control.

FgSin3 and FgSds3 are two other main subunits of the FgRPD3L histone deacetylase complex. Ten mutations identified in *FgSIN3* include eight non-sense or frameshift mutations and two missense mutations (Fig 6C). Because four nonsense mutations occured in the N-terminal region of *FgSIN3* (Fig 6C), we attempted multiple times to generate the *Fgsin3* deletion mutant. Unfortunately, no *Fgsin3* mutant was identified after screening transformants from repeated transformations, suggesting that *FgSIN3* is an essential gene in *F*. *graminearum*. Based on published RNA-seq data [25, 31], we found that *FgSIN3* has two transcripts with alternative transcript initiation sites (Fig 9A). The nonsense mutations in the N-terminal region of *FgSIN3* affected the translation of the longer transcript but had no effect on the translation of the shorter transcript. The FgSin3 protein translated from the shorter transcript has the intact histone deacetylase interacting (HID) domain (PF08295) and C-terminal (CD) domain (PF16879) (Fig 9A).

The *FgSIN3* gene also had three nonsense mutations that resulted in the truncation of the CD domain (Fig 6C). To verify the suppressive effect of mutations, we used the gene replacement approach to delete the C-terminal 180 amino acid residues (CT, 1326–1505 aa) of *FgSIN3* (Fig 9A) to mimic the nonsense mutation at W1326 in suppressor S18. The resulting *FgSIN3*^ΔCT^ transformant SIS18 (Table 1) was defective in vegetative growth, suggesting the importance of the C-terminus for FgSin3 function (Fig 9B). We then deleted the *FNG1* gene in the *FgSIN3*^ΔCT^ transformant SIS18. The resulting *fng1 FgSIN3*^ΔCT^ mutant (Table 1) was partially rescued in the growth defect of *fng1* (Fig 9B) and increased in the H4 acetylation level (Fig 9C), confirming the suppressive effect of truncation of the C-terminal 180 aa of *FgSIN3* on the *fng1* mutant.

### Null mutation in Rpd3L-specific subunit FgSds3 is suppressive to the *fng1* mutant

**Fig 10:**
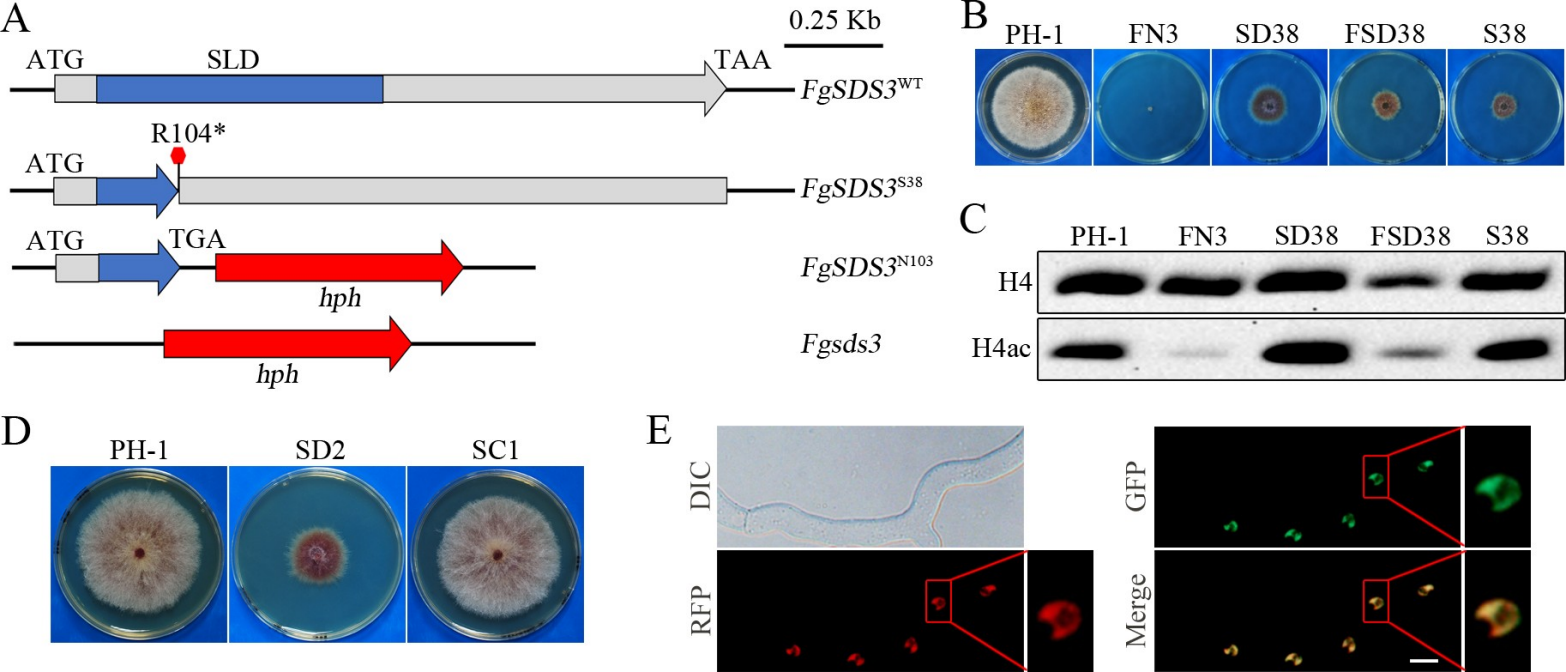
Null mutations in *FgSDS3* suppress the growth defect of the *fng1* mutant. **(A).** Schematic drawing of different alleles of *FgSDS3*. In suppressor strain S38, the nonsense mutation at R104 resulted in the truncation of part of the SLD (sds3-like domain) and the rest of FgSds3 protein. While the entire *FgSDS3* gene was deleted in the *Fgsds3* deletion, only the region after R104 was replaced with the *hph* cassette in the *FgSDS3*^N103^ mutant. (**B).** Three-day-old PDA cultures of PH-1, FN3, *FgSDS3*^N103^ (SD38), *fng1 FgSDS3*^N103^ (FSD38), and suppressor strain S38. (**C).** Western blots of total proteins isolated from the indicated strains were detected with an anti-H4ac antibody. Detection with the anti-H4 antibody was used as a loading control. (**D).** Three-day-old PDA cultures of the wild type (PH-1), *Fgsds3* mutant (SD2), and *Fgsds3*/*FgSDS3* transformant (SC1). (**E).** Germlings of the *FgSDS3*-GFP *FgRPD3*-RFP transformant (SCRR1) were examined by DIC and epifluorescence microscopy. The lower panels are close-up view of the indicated nuclei. Bar, 5 μm.

Among the nine suppressor strains with mutations in *FgSDS3*, six (S24, S26, S31, S33, S36 and S46) had frame shift mutations and one (S38) had a C^310^GA to TGA nonsense mutation at residue R104 (Fig 6D) that disrupted the Sds3-like domain (SLD) (PF08598). To investigate the relationship between Fng1 and FgSds3, we first used the gene replacement approach to generate the *FgSDS3*^N103^ transformant SD38 (Table 1) in which all but the N-terminal 104 amino acid residues were deleted (Fig 10A) to mimic the nonsense mutation in suppressor strain S38. The *FgSDS3*^N103^ mutant (Table 1) was reduced in growth in comparison with the wild type and had abnormal colony morphology (Fig 10B). We then deleted the *FNG1* gene in the *FgSDS3*^N103^ mutant SD38. The resulting *fng1 FgSDS3*^N103^ mutant FSD38 (Table 1) had similar growth defects (Fig 10B) and H4 acetylation level with suppressor strain S38 (Fig 10C), suggesting that the nonsense mutation at R104 of *FgSDS3* partially rescued the defects of the *fng1* deletion mutant.

Because the *FgSDS3*^N103^ allele has only the N-terminal 103 residues (Fig 10A), we also generated the gene replacement mutants deleted of the entire *FgSDS3* gene (Table 1, S2 Fig). The *Fgsds3* deletion mutant had similar phenotypes with the *FgSDS3*^N103^ mutant but the *Fgsds3*/*FgSDS3*-GFP complemented transformants were normal in vegetative growth (Fig 10D). Therefore, it is likely that the null mutations in *FgSDS3* are suppressive to the *fng1* mutant. In fact, all the mutations identified in *FgSDS3* in this study were nonsense or frameshift mutations. We also transformed the *FgRPD3*-RFP fusion construct into the *Fgsds3*/*FgSDS3*-GFP strain SC1 (Table 1). In the resulting transformant *Fgsds3*/*FgSDS3*-GFP *FgRPD3*-RFP (SCRR1), both FgRpd3-RFP and FgSds3-GFP signals were observed in the nucleus (Fig 10E). The overlapping distribution of FgRpd3-RFP and FgSds3-GFP signals indicated that FgSds3 likely colocalizes with FgRpd3 to the FgRpd3 HDAC complex in *F*. *graminearum*.

### Deletion of *FNG1* affects H4Ac in euchromatin and the expression of over 3,000 genes

**Fig 11:**
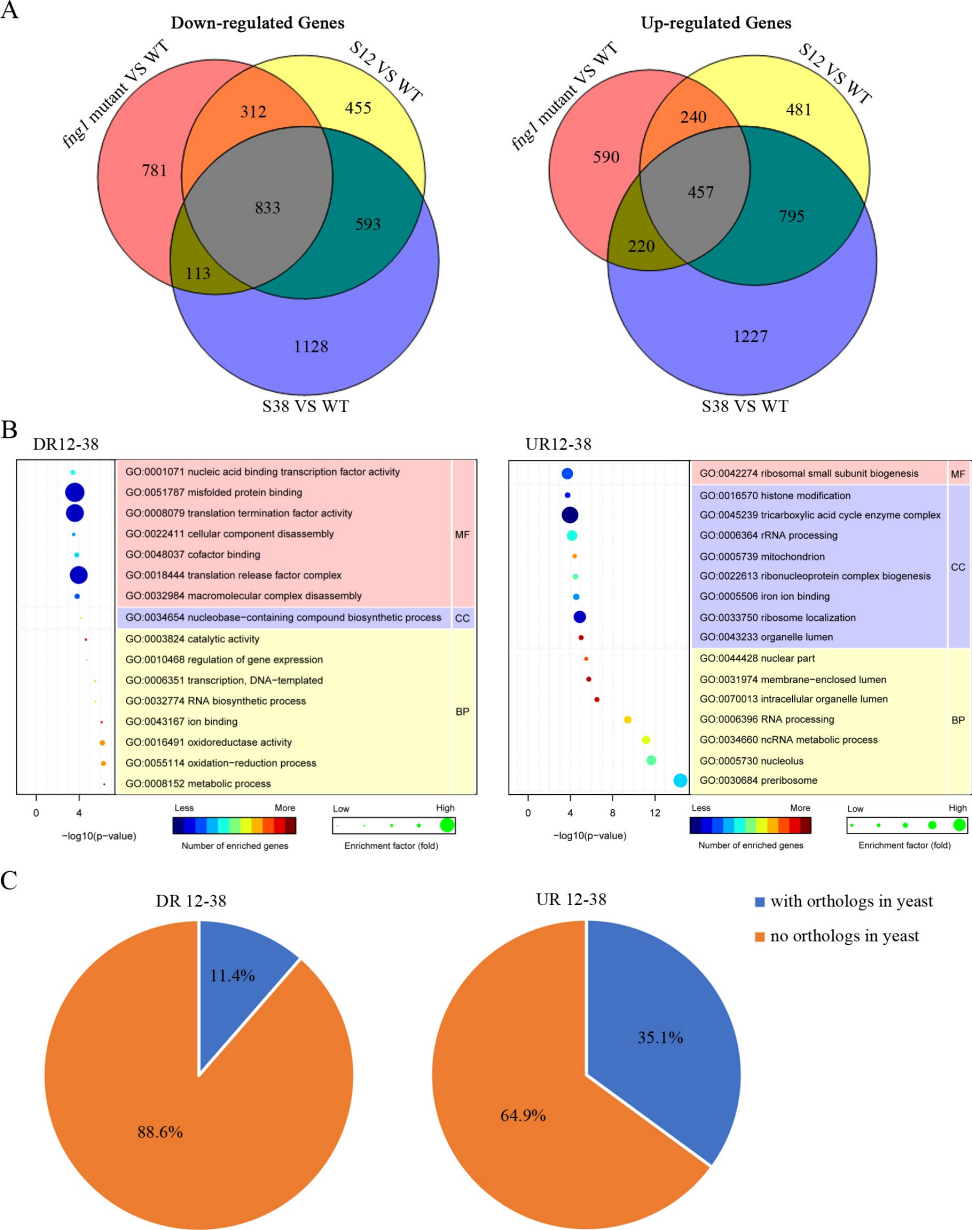
RNA-seq analysis of *fng1* mutant and suppressor strains S12 and S38. **(A).** Venn diagram showing the numbers of genes down-regulated (left panel) and up-regulated (right panel) in the *fng1* mutant, suppressor strain S12 and suppressor strain S38. (**B).** GO enrichment analysis of the down-regulated (DR12-38, left panel) and up-regulated (UR12-38, right panel) genes in *fng1* mutant and recovered in both suppressor strains S12 and S38. BP, MF, and CC stand for biological process, molecular function, and cellular component, respectively. (**C).** Pie chart showing the proportion of genes (DR12-38 and UR12-38) with and without orthologs in budding yeast.

Because histone acetylation is associated with gene expression, we used the RNA-seq approach to identify genes affected by deletion of *FNG1*. RNA samples were isolated from hyphae of the wild type and *fng1* mutant harvested from YEPD cultures at 24 h. In comparison with the wild type, 2039 differentially expressed genes (DEGs) were down-regulated over 2-fold in the *fng1* mutant (Fig 11A). The expression of 1507 DEGs, including two histone methyltransferase genes, *KMT6* [28] and *FgSET1* (FGRAMPH1_01G24837), were significantly increased in the *fng1* mutant (Fig 11A, S3 Table), indicating that Fng1 may negatively regulate their transcription. Increased transcription levels of *KMT6* and *FgSET1* likely resulted in higher H3K27 and H3K4 methylation. Interestingly, a number of genes including FGRAMPH1_01G02359, FGRAMPH1_01G02645, FGRAMPH1_01G04153, FGRAMPH1_01G11343, FGRAMPH1_01G17415, FGRAMPH1_01G18549, FGRAMPH1_01G19861, FGRAMPH1_01G26281, and FGRAMPH1_01G07387 that were specifically or highly expressed during sexual reproduction in the wild type based published RNA-seq data [31] were expressed in the hyphae of the *fng1* mutant. FGRAMPH1_01G07387 is orthologous to *Neurospora crassa SAD-3* that encodes a helicase required for ascospore development, RNAi-induced heterochromatin assembly, and meiotic silencing by unpaired DNA (MSUD) [32]. FGRAMPH1_01G02645 is the Puk1 protein kinase that plays a specific role during ascosporogenesis in filamentous ascomycetes but lacks a distinct ortholog in the budding and fission yeast [31]. The improper expression of *SAD3*, *PUK1*, and other genes that function specifically during sexual reproduction in vegetative hyphae may cause defects in hyphal growth.

To determine the relationship between genes with altered expression levels in the *Fng1* mutant and Fng1-dependent H4Ac, we compared their distribution on four chromosomes. For the 2039 down-regulated DEGs in the mutant, only 14.2% of them are in chromosomal regions enriched for Fng1-dependent H4Ac but 55.9% of them are in chromosomal regions enriched for H3K27me3 (S11 Fig). Among the DEGs up-regulated in the *fng1* mutant, 22.7% of them are in chromosomal regions deprived of Fng1-dependent H4Ac but 41.8% are in regions enriched for H3K4me2 (S11 Fig). These results suggested that many genes with altered expression in the *fng1* mutant were not directly regulated by Fng1-dependent H4Ac in *F*. *graminearum*. Defects in histone acetylation are known to indirectly regulate gene expression by affecting histone methylation or other epigenetic factors [33]. In the *fng1* mutant the upregulation of genes like *KMT6* (2.37-fold) and *FgSET1* (1.93-fold) that are important for the methylation of H3K27 and H3K4 [28] may indirectly affect gene expression in the *F*. *graminearum*.

### Mutations in suppressor strains S12 and S38 rescue the expression of many DEGS in the *fng1* mutant

To determine changes in expression profiles in spontaneous suppressors of the *fng1* mutant, RNA samples were isolated from suppressor strains S12 and S38 for RNA-seq analysis. While suppressor strain S12 had the Y248C missense mutation at a well conserved residue inside the HD domain of FgRpd3, S38 had a nonsense mutation at R104* of FgSds3. Among the genes with over 2-fold reduction in the *fng1* mutant, 781 of them had their expression increased to the wild-type levels in both suppressor strains S12 and S38 (Fig 11A). GO enrichment analysis showed that those 781 genes were enriched for genes involved in metabolic process, RNA biosynthetic process, transcription, and regulation of gene expression (Fig 11B). A number of them, such as small GTPase *RAB7* [34], isocitrate lyase *GzICL1* [35], and two cytochrome P450 monooxygenase genes (FGRAMPH1_01G04233 and FGRAMPH1_01G08139) [36], are known to be important for vegetative growth in *F*. *graminearum*. Down-regulation of these genes may directly affect hyphal growth in the *fng1* mutant.

Among the 1507 genes that were up-regulated over 2-fold in the *fng1* mutant, 590 of them had normal expression levels in suppressor strains S12 and S38 (Fig 11A). Those 590 genes are enriched for genes involved in preribosome, nucleolus, ncRNA metabolic process, RNA processing, intracellular organelle lumen, and membrane-enclosed lumen (Fig 11B). The recovery to their normal expression levels for genes with detrimental effects due to overexpression in suppressor strains S12 and S38 may also contribute to the partial rescue of growth. For examples, FGRAMPH1_01G11303 and FGRAMPH1_01G09871 orthologous to yeast *ERG6* and *SRS2*, respectively, had the wild-type expression level but upregulated over 2-fold in the *fng1* mutant. In *S*. *cerevisiae*, overexpression of *SRS2* [37] or *ERG6* [38] is detrimental to cell growth. Interestingly, 88.6% of the genes that were down-regulated in the *fng1* mutant but recovered to normal expression in both S12 and S38 have no homologs in the budding yeast (Fig 11C). Even for the genes up-regulated in the *fng1* mutant, 64.9% of them with normal expression in S12 and S38 have no homologs in *S*. *cerevisiae*. Therefore, the majority of those genes co-regulated by Fng1 and the Rpd3 HDAC complex with opposing functions appear to be unique to *F*. *graminearum* and other filamentous fungi.

Furthermore, in comparison with the wild type, 593 genes were down-regulated in both S12 and S38 but their expression was not affected in the *fng1* mutant. We also found 795 genes that were up-regulated in both S12 and S38 but not affected in the *fng1* mutant. These results suggested that the Rpd3 HDAC complex may play an important role in regulating the expression of those 1388 genes. However, Fng1 and H4 acetylation are not involved in their transcriptional regulation. On the other hand, we also found that suppressor mutations in S12 and S38 had no effects on the expression of 1290 genes with altered expression levels (over two-fold) in the *fng1* mutant (Fig 11A). These results indicate that although Fng1 and the Rpd3 HDAC complex have opposing regulatory effects on H4 acetylation and the expression of 1371 genes, they have distinct functions on regulating other subsets of genes.

## Discussion

In *S*. *cerevisiae* and *C*. *albicans*, Yng1 and Yng2 are two paralogs belonging to the NuA3 and NuA4 HAT complexes, respectively, likely generated by ancestral whole genome duplication events. Fng1, the ortholog of yeast Yng2 in *F*. *graminearum*, had no detectable interaction with NuA3 HAT FgSas3 in co-IP assays, and the *fng1* deletion mutant was normal in H3 acetylation. In contrast, the interaction between Fng1 and NuA4 histone acetyltransferase Eas1 was detected by co-IP assays, indicating that Fng1 may function as a subunit of the NuA4 HAT complex. However, it worth noting that the strains used for co-IP assays had the transforming *FNG1*-GFP, *FgSAS3*-FLAG, and *FgESA1*-flag constructs integrated ectopically in the genome. Expression from the endogenous alleles of these genes may compete with proteins expressed from the epitope-tagged alleles and interfere with their interactions in co-IP assays. Nevertheless, reduced acetylation levels of H4K5, H4K8, H4K12, and H4K16 were detected in the *fng1* mutant, suggesting that Fng1 is important for H4 acetylation, which is consistent with reduced acetylation levels of H4K5, H4K8, and H4K12 in the *S*. *cerevisiae yng2* and *Schizosaccharomyces pombe png1* mutants [12, 39]. The H4K16 acetylation level was not assayed in the *S*. *cerevisiae yng2* and *S*. *pombe png1* mutants. However, CaYng2 in *C*. *albicans* was not required for H4K16 acetylation [40]. These observations suggest that the role of Fng1 orthologs in H4K16 acetylation is not conserved among different fungi. Although the role of H4 acetylation has not been characterized in *F*. *graminearum*, H4 acetylation is known to be important for DNA double-strand break repair, cell cycle progression, and mitotic and meiotic progression in *S*.*cerevisiae* [10, 12, 41]. It is also important for genome stability, DNA damage response, and growth regulation in *S*. *pombe* [39, 42], and filamentous growth and stress responses in *C*. *albicans* [43].

In *F*. *graminearum*, the *fng1* mutant had severe growth defects. In *C*. *albicans*, the *Cayng2* deletion mutant is defective in hyphal morphogenesis [40]. The orthologs of Fng1 in *S*. *cerevisiae* and *S*. *pombe* also are involved in cell growth regulation [14, 39]. It is likely that Fng1 orthologs have a conserved role in fungal growth. Fng1 orthologs are well conserved in other plant pathogenic ascomycetes although none of them has been functionally characterized. In *F*. *graminearum*, the *fng1* mutant is non-pathogenic and fails to cause typical FHB symptoms on the inoculated kernels. Although the defects of the *fng1* mutant in growth and DON production may contribute to its loss of pathogenicity, a number of *F*. *graminearum* mutants (such as *Gzc2h088* and *Gzmyb016* deletion mutants) with severe growth defects and the *tri5* mutant blocked in DON biosynthesis still cause FHB symptoms on inoculated kernels [44, 45]. In addition, we found that infection cushion formation and infectious hyphae differentiation, two important stages in wheat infection, were impaired in the *fng1* deletion mutant. Therefore, it is possible that Fng1-mediated H4 acetylation plays a role in regulating the expression of infection-related genes and infectious growth in *F*. *graminearum*.

The *fng1* mutant was not stable and produced spontaneous suppressor mutants with fast-growing sectors. Because of the complete assembly of the *F*. *graminearum* genome, mutations in spontaneous suppressor strains can be efficiently identified by whole-genome sequencing analysis [46–49]. In this study, we identified mutations in 34 suppressor strains of the *fng1* mutant. Interestingly, all but two of them had mutations in three key components of the FgRpd3 HDAC complex, *FgRPD3*, *FgSIN3*, and *FgSDS3*. In *F*. *graminearum*, orthologs of the yeast Set3, Snt2C, Hda1, Sum1-Rfm1-Hst1 HDAC complexes also are present and likely functionally related to histone acetylation. However, besides the transducin beta-like gene *FTL1*, a key component of the Set3 HDAC complex is known to be important for plant infection and DON biosynthesis [50], other HDAC complexes remain to be characterzed in *F*. *graminearum*. Because 32 suppressor strains had mutations in the Rpd3 HDAC complex and none of the four suppressor strains analyzed by whole genome sequencing had mutations in other HDAC complexes, the opposing effects of Fng1 on H4 acetylation appeared to be specific with the Rpd3 HDAC complex.

For suppressor mutations identified in *FgRPD3*, all missense mutations occur in the HD domain, which may affect its deacetylase activity. In contrast, the frame-shift mutations are in the C-terminal TFα domain of FgRpd3. Because of the severe growth defects of the *Fgrpd3* deletion mutant, it is likely that these mutations resulting in the truncation of its C-terminal region reduced but not abolished its HDAC activities. Unlike those in *FgRPD3*, all the suppressor mutations in *FgSDS3* are nonsense or frame-shift mutations. The *FgSDS3*^N103^ mutant had similar defects with the *Fgsds3* deletion mutant, indicating that these suppressor mutations may result in null alleles of *FgSDS3*. However, *FgSIN3* is likely an essential gene because we failed to generate the *Fgsin3* deletion mutant. We also identified two missense mutations, E810K and R895H, that are in the histone deacetylase-interacting domain (HID) and the linker between the HID and CD domain of FgSin3. These two residues are well conserved among its orthologs from other fungi and the E810K or R895H mutations may affect the interaction of FgSin3 with FgRpd3 and affect its HDAC activities.

Fng1 interacts with FgEsa1 and the function of Fng1 is closely related to H4 acetylation. Although the relationship between NuA4 and Rpd3 complexes in filamentous fungi has not been reported, mutations in Esa1 catalytic subunit of the NuA4 complex recover the silencing phenotype associated with *RPD3* disruption in yeast [51]. The requirement for yeast Bmp1, an Esa1-interacting protein, could be bypassed by the inactivation of Rpd3 or Sin3 [52]. Disrupting *SDS3* rescues the nucleotide excision repair defects associated with loss of *ESA1* [53, 54]. These observations suggest that mutations in the components of the Rpd3 complex can suppress mutations related to H4 acetylation in *S*. *cerevisiae*. In this study, our data showed that mutations in FgRpd3, FgSin3, and FgSds3 were suppressive to the defects of the *fng1* mutant in growth and H4 acetylation. Although their physical associations remain to be verified in *F*. *graminearum*, suppressive effects of mutations in these genes on the *fng1* mutant suggest a functional relationship between the NuA4 HAT and Rpd3 HDAC complexes, which may be conserved in fungi or other filamentous ascomycetes, and it will be important to characterize the underlying mechanisms.

Histone acetylation is tightly associated with gene expression. Deletion of *FNG1* resulted in decreased expression of more than two thousand genes, including a number of genes known to be important for vegetative growth in *F*. *graminearum*. In contrast, a number of genes that are specifically expressed during sexual reproduction were expressed or highly induced in vegetative hyphae of the *fng1* mutant. The disordered expression of those stage-specific genes may be responsible for defects in hyphal growth. However, when RNA-seq data were compared with ChIP-seq data, the distribution of genes up- or down-regulated in the *fng1* mutant did not fully corelate with Fng1-depenent H4Ac (S11 Fig). In fact, 54.6% of DEGs down-regulated in the *fng1* mutant are in chromosomal regions depleted of H4Ac but enriched for H3K27me3, suggesting an indirect effect of *FNG1* deletion on gene expression. However, approximately 40% of the genes with altered expression in the *fng1* mutant were recovered to the wild-type expression level in both suppressor mutants S12 and S38, which may be related to growth recovery in the *fng1* mutant by suppressor mutations in the Rpd3 HDAC complex. Nevertheless, the expression of more than one-third of Fng1-regulated genes were not recovered in suppressor strain S12 or S38. Furthermore, a subset of genes had altered expression in S12 and S38 but their expression levels were normal in the *fng1* mutant, suggesting that Fng1 and the Rpd3 HDAC complex play distinct roles in regulating the expression of those genes. Interestingly, most of the genes co-regulated by Fng1 and the Rpd3 complex (with opposing functions) have no homologs in the budding yeast. Besides those genes may be involved in hyphal growth that differs from yeast budding, they may be enriched in chromosomal regions with high genetic variations and unique genes in *F*. *graminearum* [23].

Although all of the suppressor strains grew faster than the *fng1* mutant, only three mutations occurring in FgRpd3, including two adjacent missense (Y248C and F252I) and one frame-shift mutation, partially rescued the sexual reproduction defect in the *fng1* mutant to form sterile perithecia. The other suppressor strains still failed to produce perithecia. One possibility is that all of those suppressor strains were isolated because they grew faster than the original *fng1* mutant. Suppressor mutations identified in this study affected only a specific subset of genes important for hyphal growth. Another possibility is that suppressor mutations in *FgRPD3*, *FgSIN3*, or *FgRPD3* may disrupt their functions in sexual development. In addition, the functional relationship between the FgRpd3 HDAC and NuA4 HAT complexes in chromatin modifications and transcription regulation may be different between vegetative growth and sexual development. Although the *fng1* mutant is non-pathogenic, some suppressor strains cause symptoms on the inoculated kernels, but none of them could spread to neighboring spikelets, suggesting that the initial infection and spreading of invasive hyphae also are subjected to different epigenetic regulation related to H4 acetylation in *F*. *graminearum*.

Although 32 out of the 34 suppressors had mutations in the key components of the Rpd3 HDAC complex, two of them had mutations in FGRAMPH1_01G22839 that encodes a hypothetical protein without homologs in the budding and fission yeasts. However, its orthologs are well conserved in filamentous ascomycetes although none of them have been functionally characterized. The 1484-aa protein encoded by FGRAMPH1_01G22839 has 269 (18.1%) glutamine residues (Q) but lacks any known domain or motif [55]. High glutamine contents likely cause protein aggregation and glutamine-rich domains may mediate protein–protein interactions [56]. Therefore, the glutamine-rich FGRAMPH1_01G22839 protein may be a novel component of the RPD3 HDAC complex in *F*. *graminearum*, and possibly in other filamentous fungi. Interestingly, among the known components of the yeast Rpd3 complexes, the *F*. *graminearum* genome lacks the orthologs for two of them. Therefore, *F*. *graminearum* and other filamentous ascomycetes may differ from *S*. *cerevisiae* in the components of the Rpd3 HDAC complex. It will be important to further characterize the roles of FGRAMPH1_01G22839 in the FgRpd3 complex, H4 acetylation, and suppression of the *fng1* mutant.

## Materials and methods

### Identification of *FNG1*, *FgSAS3*, *FgESA1*, *FgRPD3*, *FgSIN3*, and *FgSDS3* in *F*. *graminearum*

The protein sequences of yeast Yng1 (YOR064C), Yng2 (YHR090C), Sas3 (YBL052C), and Esa1 (YOR244W) were obtained from the *Saccharomyces* genome database (www.yeastgenome.org) and used to search against the genome database of *Fusarium graminearum* strain PH-1 (RR1) at EnsemblFungi (fungi.ensembl.org/index.html) by BlastP. The protein sequences of *F*. *graminearum* homologs were then used as queries to search against the *Saccharomyces* genome database for verification. Protein domains were analyzed with Pfam (www.pfam.xfam.org).

### Strains and culture conditions

The wild-type *F*. *graminearum* strain PH-1 [23] and mutant strains generated in this study were routinely cultured on potato dextrose agar (PDA). PDA cultures grown at 25°C for 3 days were used to assay the average growth per day and colony morphology. Conidiation was determined with conidia harvested from carboxymethyl cellulose (CMC) cultures as described [57]. For sexual reproduction, aerial hyphae on carrot agar cultures were pressed down with 0.1% Tween 20 for self-fertilization and cultured at 25°C under black light (330-400nm). Perithecium formation was examined at 8 days post-fertilization (dpf) [58]. DON production in 7-day-old LTB (liquid trichothecene biosynthesis) cultures was measured using the Beacon DON Plate Kit (Beacon Analytical Systems, USA) [59].

### Targeted gene deletion

To generate the gene replacement construct for *FNG1* by the split marker approach, the 576-bp upstream and 608-bp downstream flanking sequences of the target genes were amplified with the primer pairs *FNG1*/1F - *FNG1*/2R and *FNG1*/3F - *FNG1*/4R (S4 Table) by polymerase chain reaction (PCR) from genomic DNA of PH-1. The resulting PCR products were ligated to the hygromycin phosphotransferase (*hph*) gene cassette under the control of *A*. *nidulans trpC* promoter by overlapping PCR and transformed into protoplasts of PH-1 as described [57]. Protoplast preparation and PEG-mediated transformation were performed as described [57]. Hygromycin B (CalBiochem, La Jolla, CA, USA) was added to the final concentration of 300 μg ml^–1^ for selection of transformants. Similar approaches were used to generate the *Fgrpd3* and *Fgsds3* deletion mutants with primers listed in S4 Table.

### Plasmid generation

For complementation assays, the full-length *FNG1* gene including the 1.2-kb promotor region was amplified with primer pair *FNG1* N/F—*FNG1* G/R (S4 Table) and co-transformed with *Xho*I-digested pFL2 (geneticin resistance) into yeast strain XK1-25 as described [60]. The resulting *FNG1*-GFP construct was verified by sequence analysis and integrated ectopically into the genome of *fng1* mutant. Transformants resistant to both hygromycin and geneticin were screened by PCR and examined for GFP signals with an Olympus BX-53 epifluorescence microscopy. The same yeast gap repair approach was used to generate the *FNG1*^ΔPHD^, *FgRPD3*-RFP, and *FgNOP1*-RFP constructs with primer pairs *FNG1* N/F—*FNG1*^ΔPHD^/R and *FNG1*^ΔPHD^/F—*FNG1* UTR/R, *FgRPD3* N/F—*FgRPD3* R/R, and *FgNOP1* N/F—*FgNOP1* R/R (S4 Table). The *FNG1*^ΔPHD^ construct was transformed into the *fng1* mutant to generate the complemented transformants. The *FgRPD3*-RFP, and *FgNOP1*-RFP constructs were respectively transformed into the *fng1*/*FNG1-GFP* transformant. Transformants resistant to hygromycin, geneticin, and zeocin were verified by PCR and examined for GFP and RFP signals with an Olympus BX-53 epifluorescence microscopy.

### Plant infection

Intact flowering wheat heads of cultivar Xiaoyan 22 were inoculated with PDA culture blocks. Infected wheat heads were examined for diseased spikelets at 14 days post-inoculation (dpi) to estimate the disease index. Wheat kernels at the inoculation site were collected and assayed for DON production as described [61]. To assay infection cushion formation, infected lemmas were sampled at 2 dpi, fixed with 4% (vol/vol) glutaraldehyde, and coated with gold-palladium before examination by scanning electron microscopy (SEM) as described [25]. Coleoptiles of 3-day-old seedlings of the wheat cultivar Norm were used for infection assays as described [62]. Briefly, the top 1–2 mm portion of wheat coleoptiles was excised and inoculated over the wound sites with 2 μl of freshly prepared hyphal suspensions. The seedlings were then grown at 25°C with a 12 h light/12 h dark photoperiod. Necrotic lesions on leaf sheaths were stained with Alexa Fluor 488 at 3 dpi and examined with an Olympus BX-53 epifluorescence microscopy.

### Analysis of histone acetylation levels

Hyphae were harvested from 24 h YEPD (Yeast extract peptone dextrose) cultures by filtration through two layers of Miracloth (Sigma, USA) and washed with sterile distilled water. Proteins were isolated from vegetative hyphae as described [63]. For Western blot analyses, total proteins were separated on 12.5% SDS-PAGE gels and transferred to nitrocellulose membranes. Acetylation of histone H3 and H4 was detected with the anti-Histone H3ac (K9+K14+K18+K23+K27) (ab47915), anti-Histone H4ac (K5+K8+K12+K16) (ab177790), anti-Histone H4K5ac (ab51997), anti-Histone H4K8ac (ab15823), anti-Histone H4K12ac (ab46983), anti-Histone H4K16ac (ab194352), and anti-Histone H2AK5ac (ab45152) antibodies from Abcam (Cambridge, UK). Detection with the anti-Histone H3 (ab209023, Abcam), anti-Histone H4 (ab10158, Abcam), and anti-Histone H2A (ab188312, Abcam) antibodies was used as the loading controls.

### Co-immunoprecipitation assays

To generate the *FgSAS3*-FLAG fusion construct, a *FgSAS3* fragment containing its entire open reading frame (ORF) and native promoter was amplified with primers *FgSAS3* N/F and *FgSAS3* FLAG/R (S4 Table) and cloned in pFL7 (geneticin resistance) by the yeast gap repair approach [60]. The *FgSAS3*-FLAG fusion construct recovered from yeast transformants was verified by sequence analysis and transformed individually or co-transformed with *FNG1*-GFP into the wild-type strain PH-1 to generate the *FgSAS3*-FLAG and *FgSAS3*-FLAG *FNG1*-GFP transformants. Similar approaches were used to generate the *FgESA1*-FLAG construct that was transformed alone or together with *FNG1*-GFP into PH-1 to generate the *FgESA1*-FLAG and *FgESA1*-FLAG *FNG1*-GFP transformants. Total proteins were isolated from the resulting transformants as described [63] and the expression of transforming constructs was verified by western blot analysis with the anti-GFP (11814460001, Roche, USA) and anti-FLAG (F9291, Sigma, USA) antibodies. For co-IP assays, total proteins were incubated with anti-GFP affinity beads (SA070001, Smart-lifesciences, China) for 4 h at 4°C. After washing twice, proteins bound to anti-GFP affinity beads were eluted as described [63]. Western blots of total proteins and proteins eluted from anti-GFP affinity beads were detected with the anti-GFP (11814460001, Roche) and anti-FLAG (F9291, Sigma) antibodies. Detection with an anti-Tub2 β-tubulin antibody [64] was used as the loading control.

### Spontaneous suppressor strains of the *fng1* mutant and whole genome re-sequencing analysis

Fast-growing sectors of the *fng1* mutant were transferred with sterile toothpicks to fresh PDA plates. After single spore isolation, each sub-culture of spontaneous suppressors were assayed for defects in growth, conidiation, sexual reproduction and plant infection [46]. To identify mutations in selected suppressor strains, DNA isolated from 24 h hyphae were sequenced by Illumina HiSeq-PE150 at Novogene Bioinformatics Institute (Beijing, China) to 50x coverage with pair-end libraries. The sequence reads were mapped onto the reference genome of strain PH-1 by Bowtie 2.23 [65, 66], and variants were called by SAMtools with the default parameters [67]. Annotation of the mutation sites was performed with Variant Effect Predictor (VEP) [68].

### Chromatin immunoprecipitation-sequencing (ChIP-seq) assays

ChIP assays were performed by Wuhan IGENEBOOK Biotechnology (www.igenebook.com) with hyphae harvested from 24 h YEPD cultures of PH-1 and *fng1* mutant. In brief, 1 g of hyphae were washed twice in cold 0.01 M PBS buffer (pH 7.4), cross-linked with 1% formaldehyde for 10 min at room temperature, and then quenched by the addition of glycine to the final concentration 125 mmol/L [28]. The resulting samples were resuspended in lysis buffer (0.1% SDS, 1% Triton X-100, 2 mM EDTA, 20 mM Tris-HCl pH 8.0 and 150 mM NaCl) and sonicated as described [28] to obtain soluble sheared chromatin (average DNA length of 200–500 bp). After immunoprecipitation with an anti-H4ac antibody (Cat# 39026, active motif, USA), DNA was extracted and used to construct sequencing libraries with the INEXTFLEX ChIP-Seq Library Prep Kit (NOVA-514120, Bioo Scientific, USA), and sequenced on Illumina Xten. After filtering out low-quality reads with Fastp [69], clean reads were mapped to the *F*. *graminearum* genome by Bowtie 2 [65]. The Picard toolkit was used to remove potential PCR duplicates (broadinstitute.github.io/picard/). The deepTools was used to turn BAM files of aligned reads into bigWig files which could be displayed in Integrative Genomics Viewer (IGV) [70, 71].

### Quantitative reverse transcription-polymerase chain reaction (qRT-PCR) assays

RNA samples of the wild type and *fng1* mutant were isolated from hyphae of 3-day-old LTB cultures with the Eastep Super Total RNA Extraction Kit (Promega, USA). The FastKing RT Kit (TIANGEN, China) was used to synthesize cDNA and qRT-PCR assays were performed with the CFX96 Real-Time System (Bio-RAD, USA) [72]. Relative expression levels of *TRI* genes were assayed by qRT-PCR with primers listed in S4 Table using the *F*. *graminearum* actin gene FGRAMPH1_01G24551 as the internal control [73].

### RNA-seq analysis

Hyphae of PH-1, the *fng1* mutant, and two suppressor strains S12 and S38 were harvested from YEPD cultures at 24 h and used for RNA extraction with TRIzol (Invitrogen, USA). RNA-seq libraries were prepared with the NEBNext Ultra Directional RNA Library Prep Kit (NEB, USA) following the manufacturer’s instructions and sequenced with Illumina HiSeq 2500 with the paired-end 2 × 150 bp model at the Novogene Bioinformatics Institute (Beijing, China). For each sample, at least 24 Mb of paired-end reads were obtained. The resulting RNA-seq reads were mapped onto the reference genome of *F*. *graminearum* strain PH-1 [23, 66] by HISAT2 [74]. The number of reads (count) mapped to each gene were calculated by featureCounts [75]. Differential expression analysis of genes was performed using the edgeRun package [76] with the exactTest function. Genes with log_2_FC (log_2_ fold change) greater than 1 and FDR less than 0.05 were regarded as differentially expressed genes. GO enrichment analysis was performed with Blast2GO [77]. The *P*-values were adjusted with the Benjamini-Hochberg procedure [78] by controlling false discovery rate (FDR) to 0.05. All the Perl, R, and Shell scripts used in this study for sequencing and other analysis were available on GitHub as described [79].

## Supporting information

S1 Fig Schematic drawing of the Fng1, Yng1, and Yng2 proteins with the inhibitor of growth (ING) and plant homeodomain (PHD) finger domains. (TIF)

S2 Fig Schematic drawing of the primers used to generate gene replacement constructs. The target gene (*FNG1*/*FgRPD3*/*FgSDS3*) and hygromycin phosphotransferase (*hph*) cassette are marked with black and red arrows, respectively. The upstream and downstream flanking sequences of each gene were amplified with primer pairs 1F/2R and 3F/4R and connected to overlapping fragments of the *hph* cassette. Knockout mutants generated by three homologous recombination events (marked with X) were screened by PCR with primer pairs 5F/6R and H850/H852 and further confirmed by PCR with primer pairs 7F/H855R and H856F/8R.
(TIF)

S3 Fig Assays for the defects of the *fng1* mutant in DON biosynthesis. DON production of the inoculated spikelets at 14 dpi with the wild type (PH-1), *fng1* mutant (FN3), and *fng1*/*FNG1* transformant (FC1).
(TIF)

S4 Fig Assays for the expression of genes involved into DON biosynthesis by qRT-PCR. Relative expression levels of the *TRI4*, *TRI5*, *TRI6*, and *TRI10* genes were assayed with RNA isolated from 3-day-old LTB cultures of wild type (PH-1) and *fng1* mutant (FN3).
(TIF)

S5 Fig Assays for the defects of *fng1* mutant in corn silks infection. Corn silks inoculated with culture blocks of the wild type (PH-1), *fng1* mutant (FN3), and *fng1*/*FNG1* transformant (FC1) were photographed at 5 dpi.
(TIF)

S6 Fig Schematic drawing of the primers used to generate the *FNG1*ΔPHD mutant allele. The *FNG1*^ΔPHD^ mutant allele was generated with primer pairs *FNG1* N/F—*FNG1*^ΔPHD^/R and *FNG1*^ΔPHD^/ F—*FNG1* UTR/R.
(TIF)

S7 Fig The A1195C mutation in *FgRPD3* resulted in the retention of intron 3. **(A).** The positions of labeled primers used to detect the splicing efficiency of intron 3 in *FgRPD3* transcripts. **(B).** Intron splicing efficiency assayed by RT-PCR with primers flanking intron 3 of *FgRPD3* in the wild type PH-1 and suppressor strain S29. Lanes 1–4 were PCR products amplified with cDNA, genomic DNA of suppressor strain S29 and cDNA, genomic DNA of wild type PH-1, respectively. **(C).** Retention of intron 3 in *FgRPD3* resulted frame shift mutation at Y347.
(TIF)

S8 Fig The G468A mutation occurred in *FgSDS3* resulted in retention of intron 1. **(A).** The positions of labeled primers used to detect the splicing efficiency of intron 1 in *FgSDS3*. **(B).** Intron splicing efficiency were verified by RT-PCR with primers flanking the intron 1 of *FgSDS3* in the wild type PH-1 and suppressor strain S49. Lanes 1–4 were PCR products amplified with cDNA, genomic DNA of suppressor strain S49 and cDNA, genomic DNA of wild type PH-1, respectively. **(C).** Retention of intron 1 in *FgSDS3* resulted 20 more amino-acids after K136.
(TIF)

S9 Fig Growth of suppressor mutants with mutations in *FgRPD3*, *FgSIN3* and *FgSDS3*. (TIF)

S10 Fig Two suppressor strains with missense mutations in *FgSIN3*. **(A).** Schematic drawing of FgSin3 and two missense suppressor mutations in suppressor strains S42 and S43. HID, histone deacetylase interacting domain; CD, C-terminal domain. **(B).** Four-day-old PDA cultures of suppressor strains S42 and S43.
(TIF)

S11 Fig Chromosomal distribution of differentially expressed genes (DEGs) in the *fng1* mutant in comparison with the wild type. Distribution of DEGs up- or down-regulated in the *fng1* mutant on chromosomes 1–4 of *F*. *graminearum* in comparison with sequences enriched for Fng1-dependent H4Ac (PH-1 minus the *fng1* mutant) or H3K27me3 and H3K4me2.
(TIF)

S1 Table Phenotypes of the spontaneous suppressor strains. (DOC)

S2 Table Mutations identified in suppressor strains by whole genome sequencing analysis. (DOCX)

S3 Table Expression profiles of *KMT6* and *FgSET1* in the wild type and *fng1* mutant. (DOCX)

S4 Table PCR primers used in this study. (DOC)
