# Supplementary figures and images for: Opposing functions of Fng1 and the Rpd3 HDAC complex in H4 acetylation in Fusarium graminearum
Source: PLoS Genet. 2020 Nov 2;16(11):e1009185. doi: 10.1371/journal.pgen.1009185 (PMC7660929; doi:10.1371/journal.pgen.1009185)

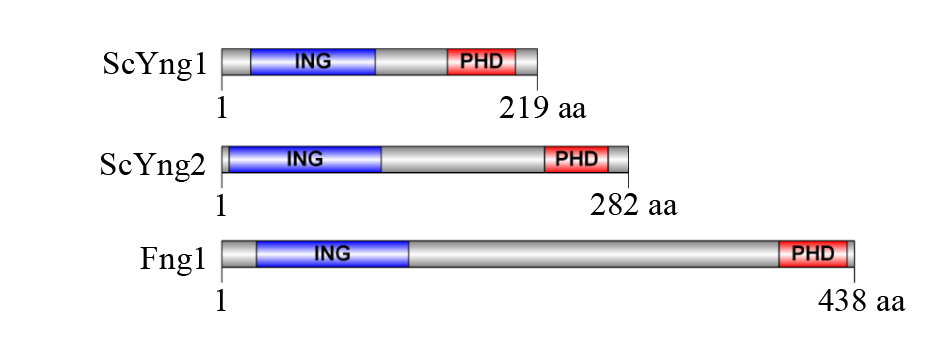

Supplement: S1 Fig — (TIF) [file pgen.1009185.s001.tif]

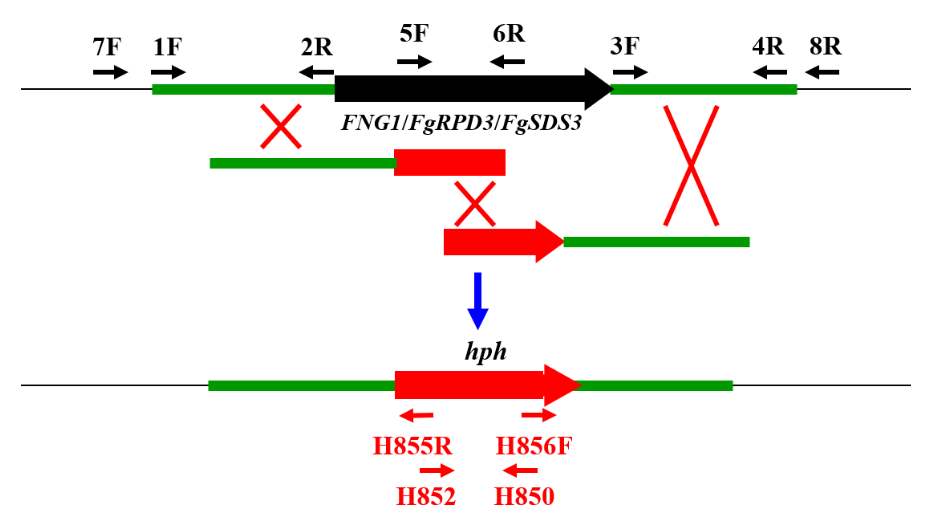

Supplement: S2 Fig — The target gene (FNG1/FgRPD3/FgSDS3) and hygromycin phosphotransferase (hph) cassette are marked with black and red arrows, respectively. The upstream and downstream flanking sequences of each gene were amplified with primer pairs 1F/2R and 3F/4R and connected to overlapping fragments of the hph cassette. Knockout mutants generated by three homologous recombination events (marked with X) were screened by PCR with primer pairs 5F/6R and H850/H852 and further confirmed by PCR with primer pairs 7F/H855R and H856F/8R. (TIF) [file pgen.1009185.s002.tif]

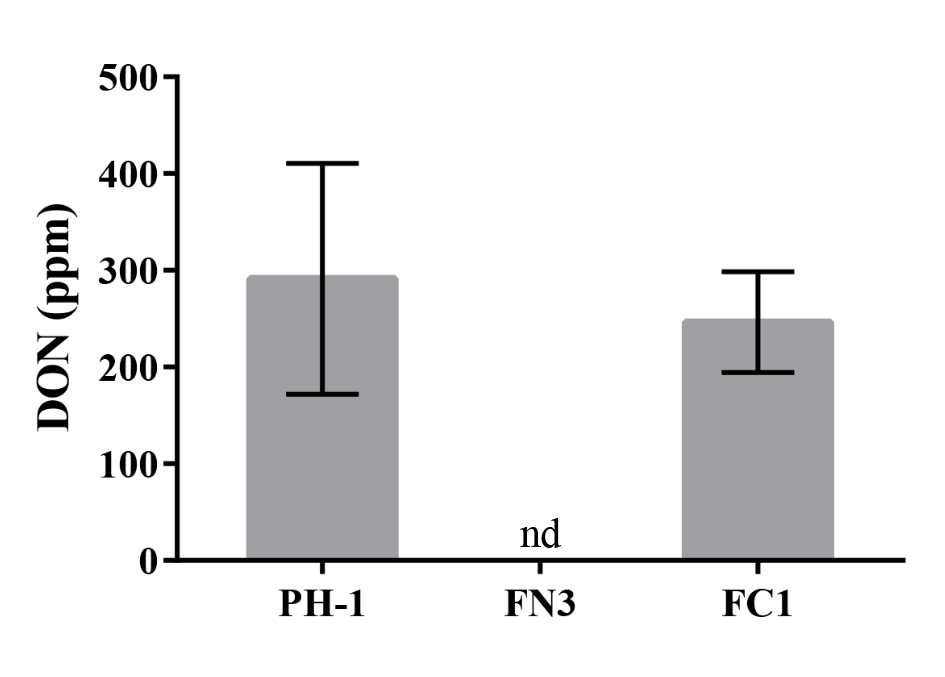

Supplement: S3 Fig — DON production of the inoculated spikelets at 14 dpi with the wild type (PH-1), fng1 mutant (FN3), and fng1/FNG1 transformant (FC1). (TIF) [file pgen.1009185.s003.tif]

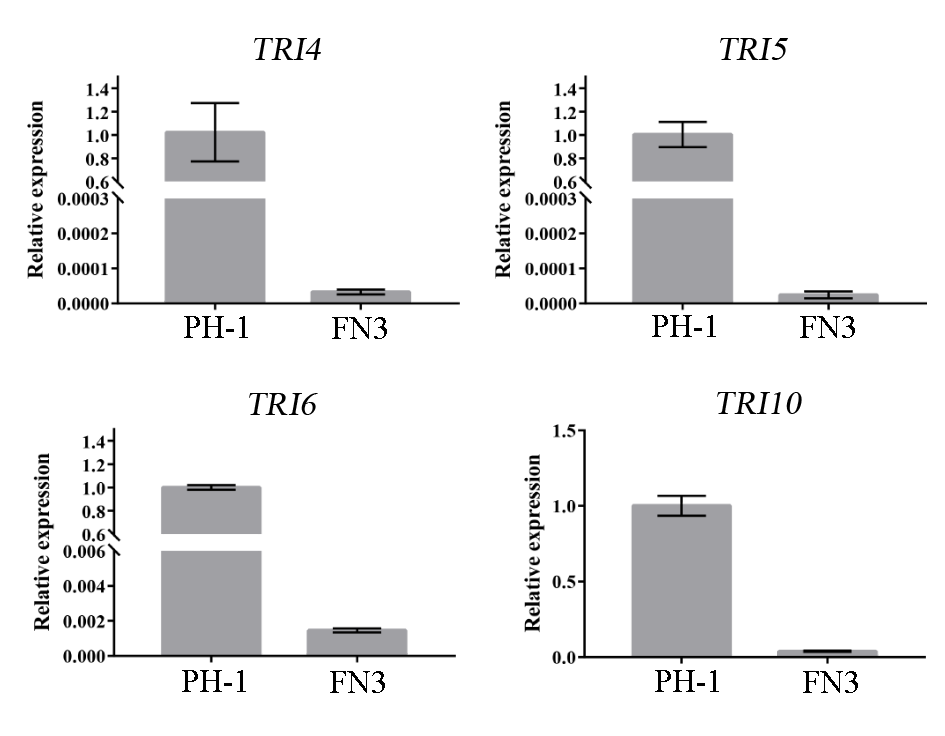

Supplement: S4 Fig — Relative expression levels of the TRI4, TRI5, TRI6, and TRI10 genes were assayed with RNA isolated from 3-day-old LTB cultures of wild type (PH-1) and fng1 mutant (FN3). (TIF) [file pgen.1009185.s004.tif]

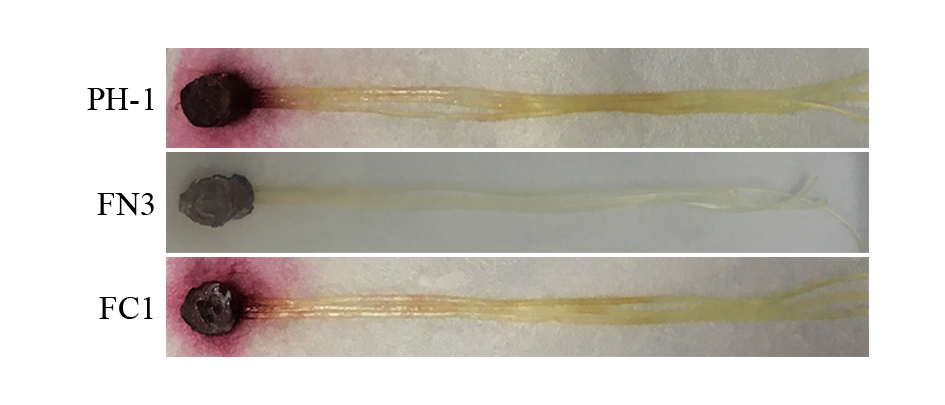

Supplement: S5 Fig — Corn silks inoculated with culture blocks of the wild type (PH-1), fng1 mutant (FN3), and fng1/FNG1 transformant (FC1) were photographed at 5 dpi. (TIF) [file pgen.1009185.s005.tif]

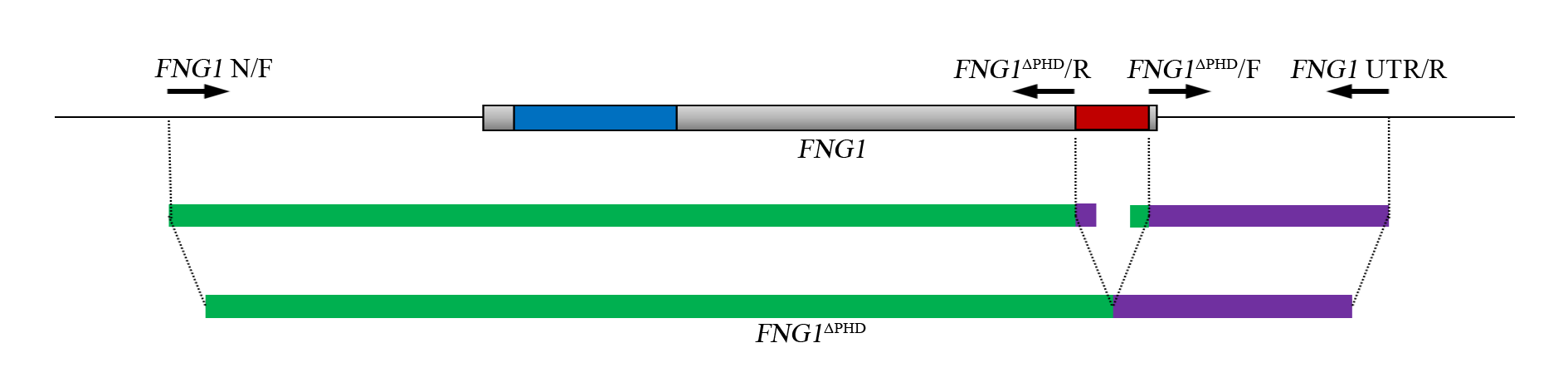

Supplement: S6 Fig — The FNG1ΔPHD mutant allele was generated with primer pairs FNG1 N/F—FNG1ΔPHD/R and FNG1ΔPHD/ F—FNG1 UTR/R. (TIF) [file pgen.1009185.s006.tif]

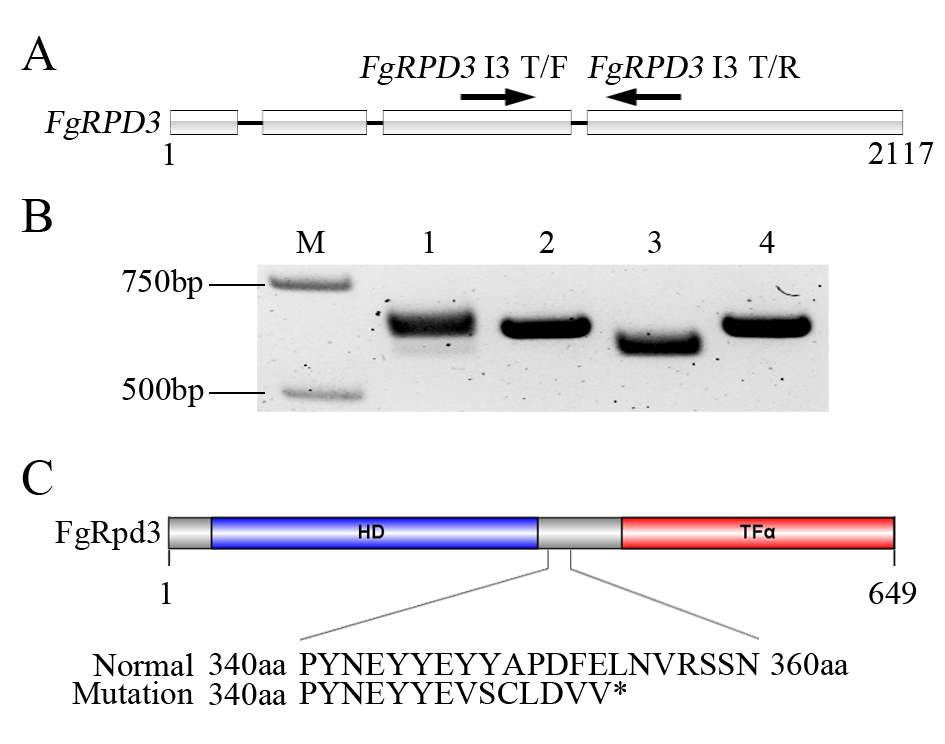

Supplement: S7 Fig — (A). The positions of labeled primers used to detect the splicing efficiency of intron 3 in FgRPD3 transcripts. (B). Intron splicing efficiency assayed by RT-PCR with primers flanking intron 3 of FgRPD3 in the wild type PH-1 and suppressor strain S29. Lanes 1–4 were PCR products amplified with cDNA, genomic DNA of suppressor strain S29 and cDNA, genomic DNA of wild type PH-1, respectively. (C). Retention of intron 3 in FgRPD3 resulted frame shift mutation at Y347. (TIF) [file pgen.1009185.s007.tif]

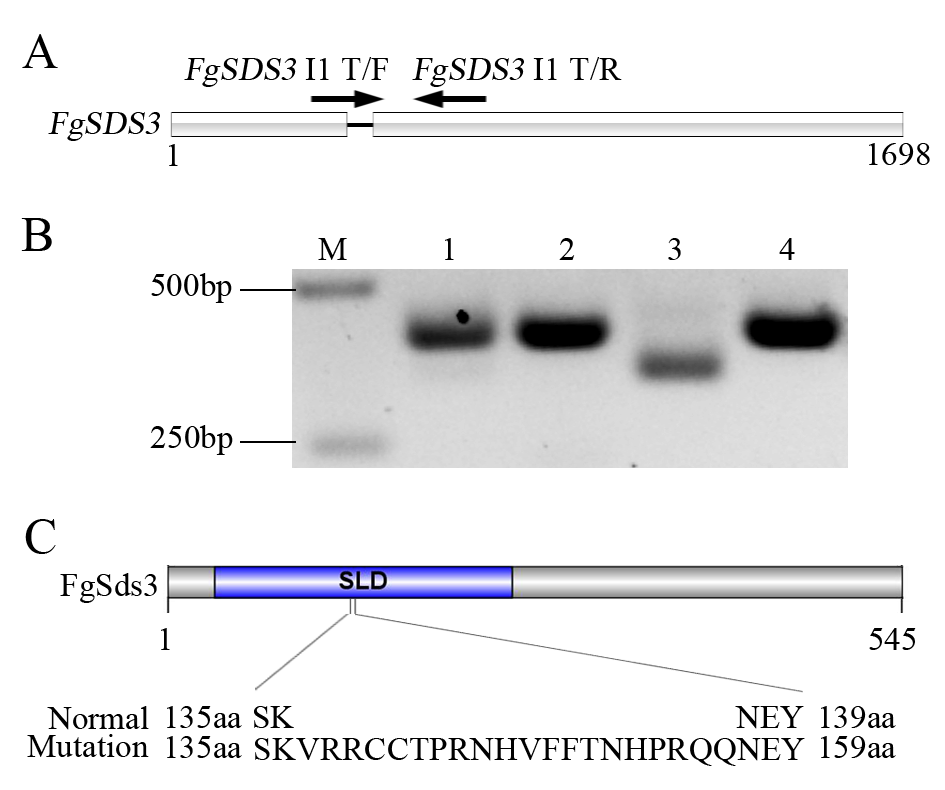

Supplement: S8 Fig — (A). The positions of labeled primers used to detect the splicing efficiency of intron 1 in FgSDS3. (B). Intron splicing efficiency were verified by RT-PCR with primers flanking the intron 1 of FgSDS3 in the wild type PH-1 and suppressor strain S49. Lanes 1–4 were PCR products amplified with cDNA, genomic DNA of suppressor strain S49 and cDNA, genomic DNA of wild type PH-1, respectively. (C). Retention of intron 1 in FgSDS3 resulted 20 more amino-acids after K136. (TIF) [file pgen.1009185.s008.tif]

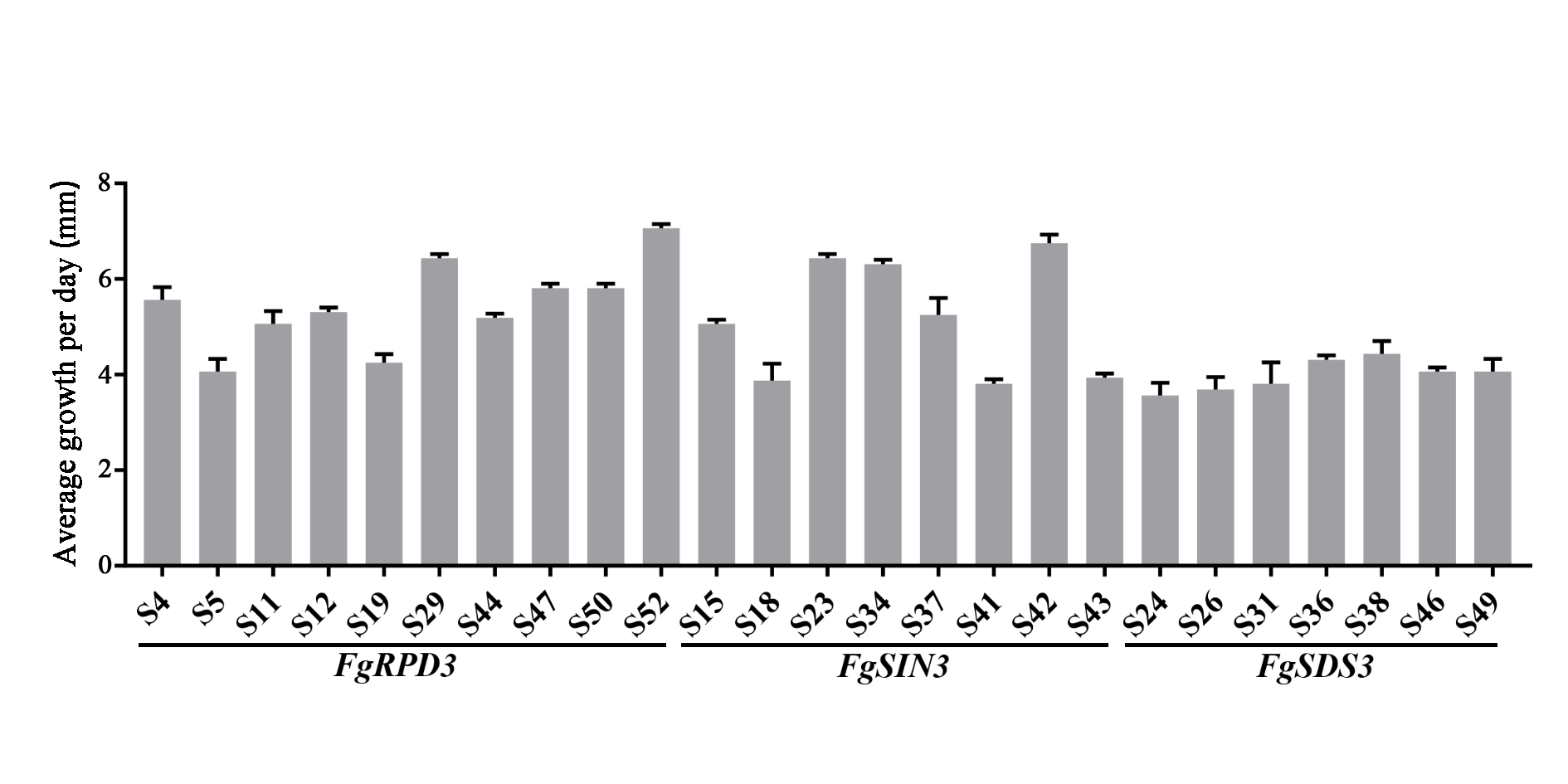

Supplement: S9 Fig — (TIF) [file pgen.1009185.s009.tif]

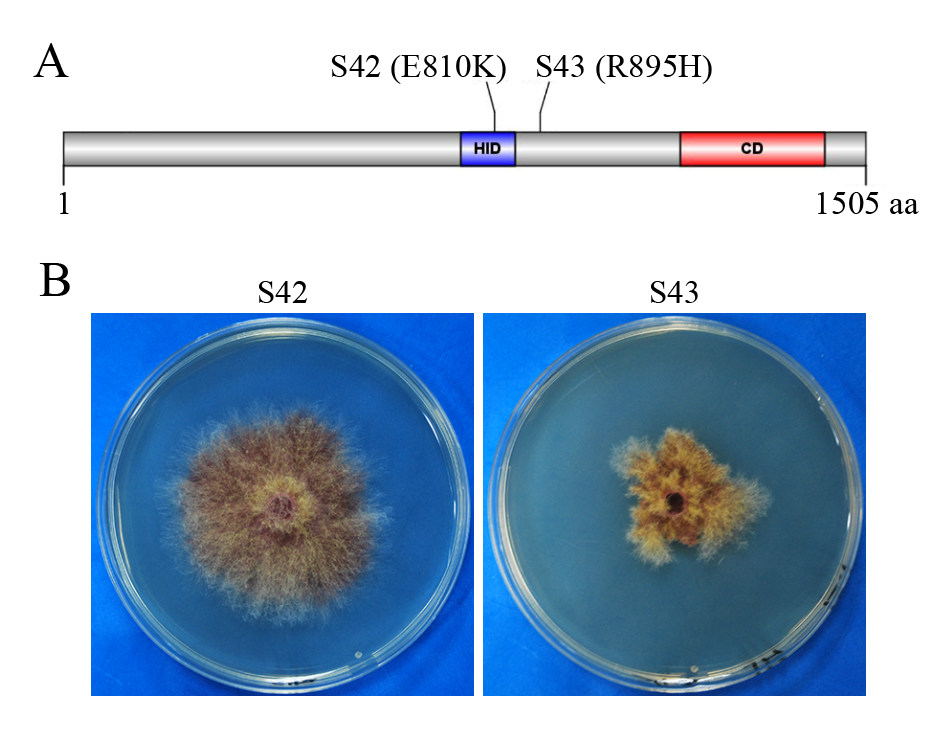

Supplement: S10 Fig — (A). Schematic drawing of FgSin3 and two missense suppressor mutations in suppressor strains S42 and S43. HID, histone deacetylase interacting domain; CD, C-terminal domain. (B). Four-day-old PDA cultures of suppressor strains S42 and S43. (TIF) [file pgen.1009185.s010.tif]

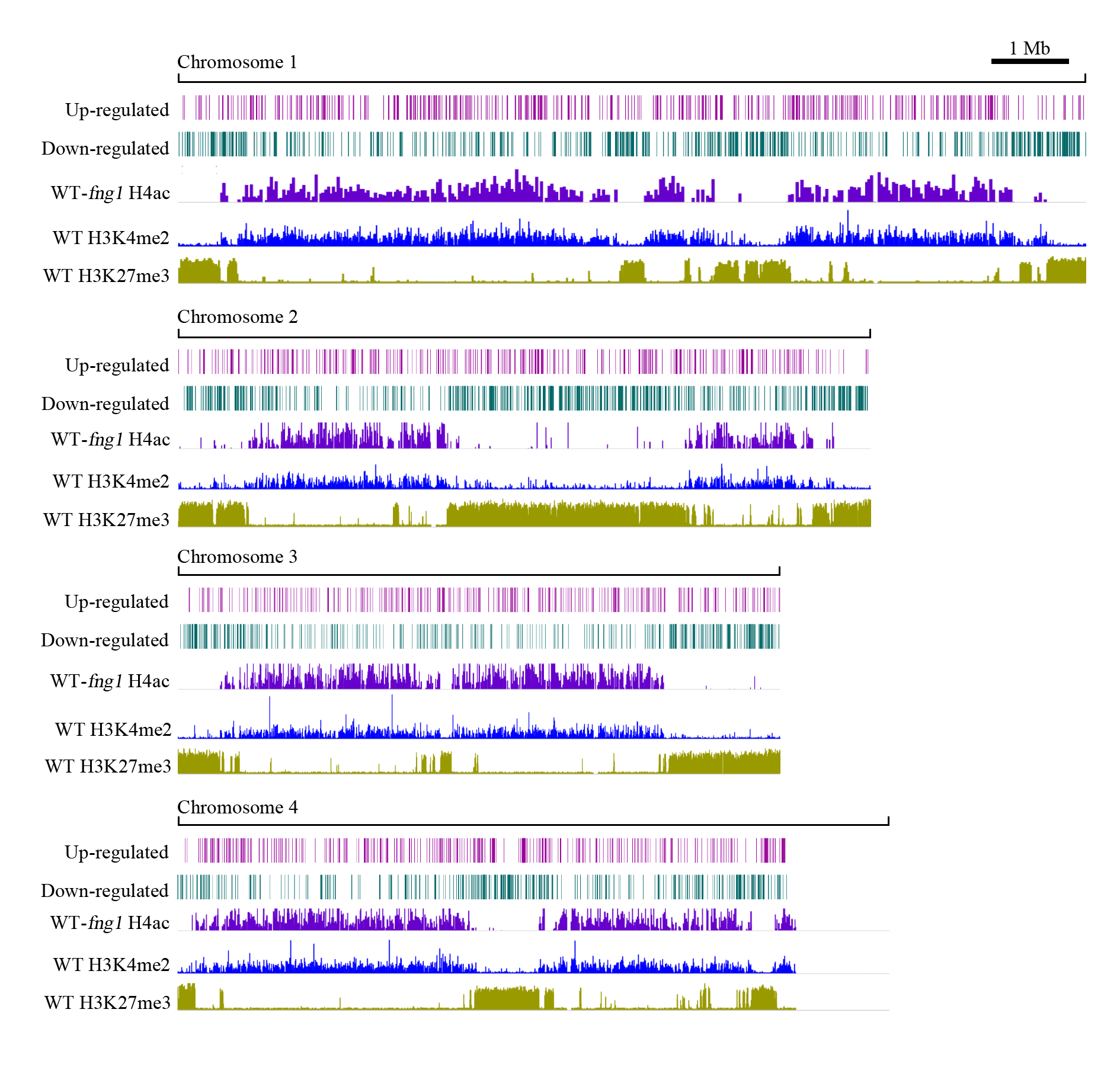

Supplement: S11 Fig — Distribution of DEGs up- or down-regulated in the fng1 mutant on chromosomes 1–4 of F. graminearum in comparison with sequences enriched for Fng1-dependent H4Ac (PH-1 minus the fng1 mutant) or H3K27me3 and H3K4me2. (TIF) [file pgen.1009185.s011.tif]
