# Supplementary material for: Opposing functions of Fng1 and the Rpd3 HDAC complex in H4 acetylation in Fusarium graminearum
Source: PLoS Genet. 2020 Nov 2;16(11):e1009185. doi: 10.1371/journal.pgen.1009185 (PMC7660929; doi:10.1371/journal.pgen.1009185)
Supplement: S1 Table — (DOC) [file pgen.1009185.s012.doc]

**S1 Table. Phenotypes of the spontaneous suppressor strains.**

| **Strain** | **Type** | **Radial growth**  **(mm/day)^a^** | **Conidiation**  **(×10^4^ spores/ml)^b^** | **Virulence ^c^** | **DON (ppm)** | **Sexual reproduction ^d^** |
| --- | --- | --- | --- | --- | --- | --- |
| S1^#^ | III | 3.1±0.4 | 1.2±0.6 | 0 | nd | Sterile |
| S4 | I | 5.6±0.2 | 14.0±3.5 | 1 | nd | Smaller perithecia, no ascospores |
| S5 | II | 4.1±0.2 | 0.3±0.2 | 0 | nd | Sterile |
| S8 | III | 2.3±0.1 | 0.8±0.3 | 0 | nd | Sterile |
| S10 | III | 2.9±0.1 | 2.0±0.5 | 0 | nd | Sterile |
| S11 | II | 5.1±0.2 | 8.2±2.4 | 0 | nd | Sterile |
| S12 | II | 5.3±0.1 | 17.7±3.9 | 1 | nd | Smaller perithecia, no ascospores |
| S15 | II | 5.1±0.1 | 10.2±4.8 | 0 | nd | Sterile |
| S16 | III | 3.4±0.1 | 17.7±1.7 | 0 | nd | Sterile |
| S18* | II | 3.9±0.3 | 12.2±1.5 | 0 | nd | Sterile |
| S19* | II | 4.3±0.1 | 1.2±0.3 | 0 | nd | Sterile |
| S22 | III | 1.4±0.1 | 0 | 0 | nd | Sterile |
| S23 | I | 6.4±0.1 | 20.7±3.6 | 1 | nd | Sterile |
| S24 | III | 3.6±0.2 | 6.0±0.8 | 1 | 0.8±0.4 | Sterile |
| S25 | III | 3.3±0.1 | 4.5±0.8 | 0 | 0.8±0.5 | Sterile |
| S26 | III | 3.7±0.2 | 9.5±1.6 | 1 | 0.5±0.3 | Sterile |
| S29 | I | 6.4±0.1 | 7.3±1.6 | 0 | nd | Smaller perithecia, no ascospores |
| S31 | II | 3.8±0.3 | 12.2±0.3 | 1 | 1.6±0.3 | Sterile |
| S32* | III | 2.4±0.1 | 0 | 0 | nd | Sterile |
| S33 | III | 3.2±0.2 | 1.5±0.8 | 0 | 0.4±0.1 | Sterile |
| S34 | I | 6.3±0.1 | 16.2±1.7 | 1 | nd | Sterile |
| S36 | II | 4.3±0.1 | 7.5±2.8 | 0 | 1.0±0.1 | Sterile |
| S37 | II | 5.3±0.3 | 5.3±2.2 | 0 | nd | Sterile |
| S38* | II | 4.4±0.2 | 11.5±2.9 | 0 | 0.6±0.4 | Sterile |
| S40 | III | 2.2±0.1 | 0.8±0.4 | 0 | nd | Sterile |
| S41 | II | 3.8±0.1 | 3.5±1.3 | 0 | nd | Sterile |
| S42 | I | 6.8±0.1 | 20.7±6.0 | 0 | nd | Sterile |
| S43 | II | 3.9±0.1 | NA | 0 | nd | Sterile |
| S44 | II | 5.2±0.1 | 8.0±1.0 | 0 | nd | Sterile |
| S46 | II | 4.1±0.1 | 4.3±1.8 | 0 | 1.2±0.4 | Sterile |
| S47 | I | 5.8±0.1 | 12.0±1.3 | 1 | nd | Sterile |
| S49 | II | 4.1±0.2 | 1.2±0.2 | 1 | nd | Sterile |
| S50 | I | 5.8±0.1 | 13.0±2.5 | 0 | nd | Sterile |
| S52 | I | 7.1±0.1 | 23.2±4.1 | 1 | nd | Sterile |

^#^ The numbering of suppressors were named according to the time of collection.

* indicates suppressor strains selected for were selected for genome sequencing.

^a^ Average daily extension in colony radius on PDA plates.

**^b^** Conidiation in 5-day-old CMC cultures. Mean and standard deviation were calculated from three biological replicates.

**^c^** The number of diseased spikelets on each inoculated wheat head at 14 dpi.

**^d^** Mating cultures were examined for perithecium formation.

0 indicates non-pathogenic

1 indicates limited symptoms on the inoculated kernels

NA, not assayed.

nd, not detected.
