# Supplementary material for: Opposing functions of Fng1 and the Rpd3 HDAC complex in H4 acetylation in Fusarium graminearum
Source: PLoS Genet. 2020 Nov 2;16(11):e1009185. doi: 10.1371/journal.pgen.1009185 (PMC7660929; doi:10.1371/journal.pgen.1009185)
Supplement: S2 Table — (DOCX) [file pgen.1009185.s013.docx]

**S2 Table. Mutations identified in suppressor strains by whole genome sequencing analysis.**

| Suppressors | Mutation sites | Mutation locations | Amino acid changes |
| --- | --- | --- | --- |
| S18 | TG^4468^G to TAG | FGRAMPH1_01G27415 | W1326* |
| S19 | CC^407^T to CTT | FGRAMPH1_01G01959 | P112L |
|  | C to T | Intergenic sequence  downstream of FGRAMPH1_01G23127 | - |
| S32 | C^1413^AA to TAA | FGRAMPH1_01G22839 | Q431* |
| S38 | C^310^GA to TGA | FGRAMPH1_01G02071 | R104* |

* stop codon; - no change
