# Supplementary material for: Opposing functions of Fng1 and the Rpd3 HDAC complex in H4 acetylation in Fusarium graminearum
Source: PLoS Genet. 2020 Nov 2;16(11):e1009185. doi: 10.1371/journal.pgen.1009185 (PMC7660929; doi:10.1371/journal.pgen.1009185)
Supplement: S3 Table — (DOCX) [file pgen.1009185.s014.docx]

**S3 Table. Expression profiles of *KMT6* and *FgSET1* in the wild type and *fng1* mutant.**

| Gene locus number | Hyphae CPM* | | | |
| --- | --- | --- | --- | --- |
|  | WT-1 | WT-2 | *fng1*-1 | *fng1*-2 |
| *KMT6* (FGRAMPH1_01G02263) | 30.75 | 32.34 | 73.95 | 75.11 |
| *FgSET1* (FGRAMPH1_01G24837) | 25.35 | 23.09 | 47.96 | 46.16 |

* The CPM (Counts of exon model Per Million mapped reads) value from RNA-seq data of vegetative hyphae (24 h YEPD cultures).
