# Supplementary material for: Opposing functions of Fng1 and the Rpd3 HDAC complex in H4 acetylation in Fusarium graminearum
Source: PLoS Genet. 2020 Nov 2;16(11):e1009185. doi: 10.1371/journal.pgen.1009185 (PMC7660929; doi:10.1371/journal.pgen.1009185)
Supplement: S4 Table — (DOC) [file pgen.1009185.s015.doc]

**S4 Table. PCR primers used in this study.**

| **Primer** | **Sequence (5΄→3΄)** |
| --- | --- |
| *FNG1*/1F | CCGAGGCTGCTTCTTGTAT |
| *FNG1*/2R | TTGACCTCCACTAGCTCCAGCCAAGCCCGAAGCCCTCAAAAACTGT |
| *FNG1*/3F | GAATAGAGTAGATGCCGACCGCGGGTTTAGAATGCGGTCGAGTGCT |
| *FNG1*/4R | TTGGTCCTGATGTCCTTGC |
| *FNG1*/5F | CGAGGTTGCCGACAAAGAC |
| *FNG1*/6R | TCGACATCCGAAAGCTCAC |
| *FNG1*/7F | TGTAATGTGGGGAGTGCCT |
| *FNG1*/8R | TTGTCAAGGTTCTGGCTGC |
| HYG/F | GGCTTGGCTGGAGCTAGTGGAGGTCAA |
| HY/R | GTATTGACCGATTCCTTGCGGTCCGAA |
| HYG/R | AACCCGCGGTCGGCATCTACTCTATTC |
| YG/F | GATGTAGGAGGGCGTGGATATGTCCT |
| H855/R | GCTGATCTGACCAGTTGC |
| H856/F | GTCGATGCGACGCAATCGT |
| H850 | TTCCTCCCTTTATTTCAGATTCAA |
| H852 | ATGTTGGCGACCTCGTATTGG |
| *FNG1* N/F | CGACTCACTATAGGGCGAATTGGGTACTCAAATTGGAGTTATCGCGATCATCGTC |
| *FNG1* G/R | CACCACCCCGGTGAACAGCTCCTCGCCCTTGCTCACCTTGCTTTTTTTGAATTTCTCAG |
| *FgESA1* N/F | CGACTCACTATAGGGCGAATTGGGTACTCAAATTGGGTGCCGTCTCTTCAATCTTTGAGC |
| *FgESA1* FLAG/R | CTTTATAATCACCGTCATGGTCTTTGTAGTCCCATCCCCATGTTCGGCTTGAAG |
| *FgSAS3* N/F | CGACTCACTATAGGGCGAATTGGGTACTCAAATTGGAAGAAGAGCCTCAAAAGGAACGG |
| *FgSAS3* FLAG/R | CTTTATAATCACCGTCATGGTCTTTGTAGTCAATGTCCTCCCCTTCGGCATCA |
| *FNG1*^ΔPHD^/F | TGGCGACGACAAAAAATTCAAAAAAAGCAAGTAGCCTTCGTCAC |
| *FNG1*^ΔPHD^/R | CTACTTGCTTTTTTTGAATTTTTTGTCGTCGCCAGCCTCTTC |
| *FNG1* UTR/R | CACCACCCCGGTGAACAGCTCCTCGCCCTTGCTCACTTGAGACTCTCTTCTTGATCCTGGA |
| *FgRPD3* test/F | AGGTTTTCGCTGTTCCACGCA |
| *FgRPD3* test/R | CATCCGTTCAAAACTCAAGGCCC |
| *FgSIN3* test/F | CGCGCACAACAAGCATGATGTA |
| *FgSIN3* test/R | GACCATTGAGTTTCACGATAGGCG |
| *FgSDS3* test/F | CTCTCCCTCTCCTCAACCTTTCT |
| *FgSDS3* test/R | TCTGACGCTTCAGCTCATTTCTT |
| 01G22839 test/F | TCCGCTGTTTAGCTTCCCACTC |
| 01G22839 test/R | TTGTGATCCCAGGCGACGTCTT |
| *ACRIN* qPCR/F | ATCCACGTCACCACTTTCAA |
| *ACRIN* qPCR/R | TGCTTGGAGATCCACATTTG |
| *TRI4* qPCR/F | TAAACGCCCGCGAAGTTCACA |
| *TRI4* qPCR/R | TGGTGATGGTTCGCTTCGAG |
| *TRI5* qPCR/F | CAGGTGATGGACACGATTGA |
| *TRI5* qPCR/R | CTTCTTGGCGTCCTCTGTATC |
| *TRI6* qPCR/F | AAAGCGGACGGGACTTTAG |
| *TRI6* qPCR/R | CCTTTGGTGCCGACTTCTT |
| *TRI10* qPCR/F | GAGAGAGTGGCTGTTGACTATAC |
| *TRI10* qPCR/R | TTCCATACCATCGCCTGTTC |
| *FgRPD3* N/F | CGACTCACTATAGGGCGAATTGGGTACTCAAATTGGGCCGTTGCCAAGTAAACTCCTG |
| *FgRPD3* R/R | GATGGCCATGTTATCCTCCTCGCCCTTGCTCACGTTTGCTGGGCTCTTGCTCTTC |
| *FgNOP1* N/F | CGACTCACTATAGGGCGAATTGGGTACTCAAATTGGCCGTACAACAGCACATCCTCCTC |
| *FgNOP1* R/R | GATGGCCATGTTATCCTCCTCGCCCTTGCTCACGGACTTGTAGCGCAAGTACTCG |
| *FgRPD3* I3 T/F | ATGGTATCACCGATGCGTCATAC |
| *FgRPD3* I3 T/R | TGTCATGTTCAACATGCTTGTCC |
| *FgSDS3* I1 T/F | TGGCGGGTATTCGGTTTTCTGAT |
| *FgSDS3* I1 T/R | ACGTGTGGCTCGCTTGCC |
| *FgRPD3*/1F | GTGGTACCATCCGACAACCGAC |
| *FgRPD3*/2R | TTGACCTCCACTAGCTCCAGCCAAGCCGCGGTTACTGAGGGTGTCGCGAAT |
| *FgRPD3*/3F | GAATAGAGTAGATGCCGACCGCGGGTTCGATGCTATGGACGTTGACACGG |
| *FgRPD3*/4R | CACAGGCTACACAAGATCAGTGAC |
| *FgRPD3*/5F | AAGAGTTTTAACCTCCCTACC |
| *FgRPD3*/6R | TATCGTCATTCACATCGTGTC |
| *FgRPD3*/7F | TGCCCTTGACGGACTGGAT |
| *FgRPD3*/8R | CATACAACACTTACACTCACAGCC |
| *FgSDS3*/1F | GATGCGGCAGTGGTGGAAGATAC |
| *FgSDS3*/2R | TTGACCTCCACTAGCTCCAGCCAAGCCGGTTGAGGAGAGGGAGAGTCGTG |
| *FgSDS3*/3F | GAATAGAGTAGATGCCGACCGCGGGTTAGTGGCTGCCCTGTCAAGTGC |
| *FgSDS3*/4R | CAAGATGGTTACCGCGGCTGGA |
| *FgSDS3*/5F | CAGCAGGAACATACGAATACTCAGG |
| *FgSDS3*/6R | GGTTGCGAAGAATGTATTGATGCG |
| *FgSDS3*/7F | TTCACTGGTGTGGAAGGATA |
| *FgSDS3*/8R | CGTCTTATCAACGTCTGGCGAC |
| *FgSDS3* N/F | CGACTCACTATAGGGCGAATTGGGTACTCAAATTGGGATGCGGCAGTGGTGGAAGATAC |
| *FgSDS3* G/R | CACCACCCCGGTGAACAGCTCCTCGCCCTTGCTCACCTTGCGTCCCTTGCCACGG |
